# Cyclic Evolution of Synergized Spin and Orbital Angular Momenta

**DOI:** 10.1002/advs.202409377

**Published:** 2024-11-28

**Authors:** Lei Liu, Xiao‐Chen Sun, Yuan Tian, Xiujuan Zhang, Ming‐Hui Lu, Yan‐Feng Chen

**Affiliations:** ^1^ National Laboratory of Solid State Microstructures and Department of Materials Science and Engineering Nanjing University Nanjing 210093 China; ^2^ Jiangsu Key Laboratory of Artificial Functional Materials Nanjing 210093 China; ^3^ Collaborative Innovation Center of Advanced Microstructures Nanjing University Nanjing 210093 China

**Keywords:** orbital angular momentum, spin angular momentum, topology

## Abstract

Spin angular momentum (SAM) and orbital angular momentum (OAM) are fundamental physical characteristics described by polarization and spatial degrees of freedom, respectively. Polarization is a feature of vector fields while spatial phase gradient determines the OAM ubiquitous to any scalar field. Common wisdom treats these two degrees of freedom as independent principles to manipulate wave propagations. Here, their synergy is demonstrated. This is achieved by introducing two orthogonal *p*‐orbitals as eigenbases, whose spatial modal features are exploited to generate OAM, and the associated orbital orientations provide means to simultaneously manipulate polarizations. Through periodic modulation and directional coupling, a full cyclic evolution of synchronized and synergized SAM‐OAM is realized. Remarkably, this evolution acquires a nontrivial geometric phase, leading to its representation on a Möbius strip. Experimentally, an acoustic cavity array is designed, whose dipole resonances precisely mimic the *p*‐orbitals, with pressure fields featuring the spatial characteristics and velocity fields the vectorial orientations. Based on this synergy, a spin‐orbital‐Hall effect is further showcased, highlighting the intricate locking of handedness, directionality, spin density, and spatial mode profile. This study unveils a fundamental connection between SAM and OAM, promising avenues for their novel applications in trapping, coding, and communications.

## Graphical abstract

This study reveals the synergy between spin and orbital angular momenta through a cyclic evolution on a Möbius strip. *p*‐orbitals serve a dual role to simultaneously manipulate the orbital angular momenta and polarizations. This is demonstrated in an acoustic cavity array, enabling a novel spin‐orbital Hall effect showcasing the intricate interplay of handedness, directionality, spin density, and spatial mode profile.

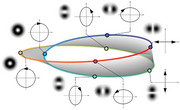

## 1 Introduction

Exploring the intricate interplay between spin (SAM) and orbital angular momentum (OAM) has been a longstanding and compelling quest in wave physics. Spin is an intrinsic form of angular momentum carried by elementary particles like electrons.^[^ ^1^ ^]^ Over a century ago, Poynting predicted a circularly polarized light carrying angular momentum,^[^ ^2^ ^]^ nowadays attributed to the ℏ spin of the photons, known as SAM. In a broader context, SAM characterizes the orientations of the electric or magnetic fields, i.e., the polarizations.^[^ ^3^ ^]^ Apart from SAM, light also carries OAM, which was identified by Allen et al. in 1992.^[^ ^4^ ^]^ They recognized that unlike SAM requiring rotations of vectors, OAM can be generated by twisted azimuthal phase gradients, which are associated with spatial degrees of freedom and thus ubiquitous in various realms of waves, including structured light,^[^ ^5^ ^]^ quantum optics,^[^ ^6^ ^]^ light manipulations based on meta‐structures,^[^ ^7^, ^8^, ^9^ ^]^ and even longitudinal waves like acoustic waves.^[^ ^10^, ^11^, ^12^ ^]^ While OAM is a common feature, it was only recently recognized that with evanescent or interference fields acoustic waves can locally hold polarizations characterized by the rotations of the velocity fields, manifested due to their vector attributes.^[^ ^13^, ^14^, ^15^, ^16^, ^17^, ^18^, ^19^, ^20^ ^]^ Generating nonzero SAM in curl‐free systems further expands approaches to understanding and manipulating waves using angular momenta.

While SAM and OAM are often treated as independent principles governing distinct degrees of freedom (SAM for the vector aspects and OAM for the spatial scalar aspects), they can interact with each other, pertaining to the so‐called spin‐orbital interactions (SOIs).^[^ ^21^ ^]^ There are primarily two types of SOIs. The first one is associated with the helicity‐dependent position or momentum for wave propagations, known as the spin‐Hall effect.^[^ ^22^ ^]^ One famous example is the classical version of the topological quantum spin‐Hall effect. Therein, the propagations of the topological edge states are found to depend on the helicity of SAM.^[^ ^23^, ^24^ ^]^ The second type of SOI concerns SAM‐to‐OAM (or OAM‐to‐SAM) conversion.^[^ ^25^, ^26^, ^27^ ^]^ Combined with the vector features of SAM, the scalar OAM waves are expanded to more general types of vectorial vortices.^[^ ^28^, ^29^ ^]^ In these vectorial states, the polarization constantly changes direction when tracing the azimuthal phase gradient, suggesting a SAM‐OAM conversion.

Historically, SAM and OAM can be independently manipulated or interact with each other. This forms the traditional understanding. However, a crucial piece has been missing—whether SAM and OAM can be synchronized or synergized. Here, we bridge this gap by demonstrating that in addition to SOIs with the essence of one angular‐momentum component affecting the other, SAM and OAM have another fundamental connection—*synergy*. Such a unique connection is established by considering two orthogonal *p*‐orbitals as eigenbases, whose spatial mode profiles contribute to generating twisted phase gradient for OAM while the associated orbital orientations control vector polarization (SAM). Through periodic modulation and directional coupling, we realize a full cyclic evolution of SAM‐OAM synergy. That is, linear‐polarization evolves into circular‐polarization and back to itself, accompanied by changes in OAM from linear momentum to a perfect vortex and back. This cyclic evolution acquires a nontrivial geometrical phase and therefore can be represented on a Möbius Strip. We experimentally verify the SAM‐OAM synergy in an acoustic cavity array where the dipole resonances act as *p*‐orbital eigenbases and the associated pressure and velocity fields exhibit the spatial OAM and the vector SAM features, respectively. Building on this synergy, we further showcase a spin‐orbital‐Hall effect, highlighting the intricate locking of handedness, directionality, spin density, and spatial mode profile. The revelation of such a fundamental and unique synergy relationship deepens the current understanding of angular momenta, introduces additional degrees of freedom to manipulate acoustic waves and can be readily extended to other wave systems with both scalar and vector components, such as water, elastic, and electromagnetic waves.

## 2 Results

### 2.1 *p*‐Orbitals and their Acoustic Realizations

**Figure 1:**
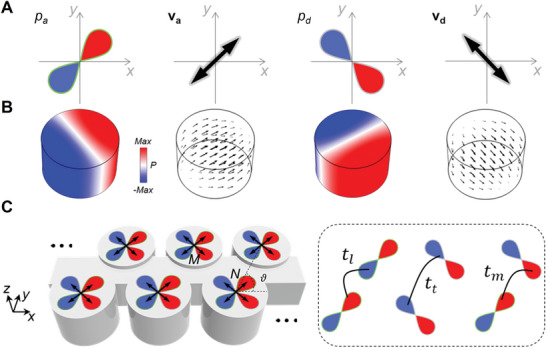
Acoustic resonances mimicking *p*‐orbitals. A) Schematics of two orthogonal *p*‐orbitals and their orientations. B) The physical realization in an acoustic cylindrical cavity supporting two dipole resonances, whose pressure and velocity fields serve as the essential spatial and vector degrees of freedom, respectively. C) A 1D lattice constructed by periodically arranging the *p*‐orbitals (or the acoustic cavities). Longitudinal, transverse, and crossed couplings between the *p*‐orbitals are illustrated in the right plane.

We start with two orthogonal *p*‐orbitals, labeled by |*p_a_* 〉 and |*p_d_* 〉, as schematically illustrated in **Figure** **1**A. Without loss of generality, the orientation of |*p_a_* 〉 (|*p_d_* 〉) is set along the antidiagonal (diagonal) direction (see the arrows in Figure 1A). In the Jones vector representation, the corresponding vector bases are $v_{a}=\frac{\sqrt{2}}{2}\;{\left[\begin{array}{cc} 1 & 1 \end{array}\right]}^{T}$ and $v_{d}=\frac{\sqrt{2}}{2}\;{\left[\begin{array}{cc} 1 & -1 \end{array}\right]}^{T}$. Orbitals are traditionally used to describe the positions and wavelike behaviors of electron clouds in an atom.^[^ ^30^ ^]^ Nowadays they are often treated as symbolic eigenbases in tight‐binding methods and have physical correspondences in a variety of systems. For example, consider an acoustic cylindrical cavity that supports two degenerate dipole resonances, as shown by the field distributions in Figure 1B (see Methods and Section S1, Supporting Information). The pressure fields precisely mimic the spatial features of the two *p*‐orbitals and the velocity fields exactly align with their orientations. Such a correspondence is inherently rooted in the incompressible linear Euler equation where the vector velocity field is connected with the gradient of the scalar pressure field.^[^ ^31^ ^]^

By carefully controlling the spatial overlap of the *p*‐orbitals and their phase difference, nonzero OAM can be generated. Such a strategy is parallel with conversing Hermite Gaussian modes to Laguerre Gaussian modes. In this way, the *p*‐orbitals serve as spatial eigenbases to manipulate OAM. Simultaneously, we exploit the orientations of the *p*‐orbitals as the vector eigenbases, whose superpositions offer means to control SAM, similar to the superpositions of linear‐polarizations producing elliptical‐ and circular‐polarizations. By harnessing both the spatial and vector features inherent in *p*‐orbitals, as opposed to isotropic *s*‐orbitals, we bring SAM and OAM into a domain where they can be concurrently manipulated, resulting in a synergistic relationship. To illustrate a full cycle of this synergy, we introduce a periodic modulation by arranging the *p‐*orbitals (or physically the acoustic cavities) into a quasi‐1D lattice along the *x*‐direction, as depicted in Figure 1C. Each unit cell comprises two sites, labeled by M and N, each hosting a pair of orthogonal *p*‐orbitals. Sites M and N are further dislocated along the *y*‐direction to introduce a directional coupling (characterized by the angle ϑ), which effectively generates distinct gauge potentials for |*p_a_* 〉 and |*p_d_* 〉, leading to a phase difference between them and therefore giving rise to nonzero OAM. Concurrent with the same phase change, the vector bases **v**_*a*_ and **v**_*d*_ are superposed, leading to nonzero SAM in synergy with the OAM. Due to the periodicity, the phase change depends on the wave vector and is periodic as well. Correspondingly, the OAM and SAM undergo a full cycle of synergy.

### 2.2 Cyclic Evolution of Synergized SAM and OAM

The proposed model is characterized by three coupling coefficients, i.e., *t_t_*, *t_l_* and *t_m_* for the transverse, longitudinal, and crossed coupling, respectively (see illustrations in Figure 1C). The Hamiltonian is written as $H\;\left(k\right)=\left[\begin{array}{cc} 0_{2\times 2} & h(k)\\ h^{†}\left(k\right) & 0_{2\times 2} \end{array}\right]$, with $h^{†}\left(k\right)=\left[\begin{array}{cc} t_{t}+t_{l}e^{ik} & t_{m}+t_{m}e^{ik}\\ t_{m}+t_{m}e^{ik} & t_{l}+t_{t}e^{ik} \end{array}\right]$, corresponding to eigenfunction $\left|\psi \right\rangle ={\left(\phi_{M,p_{a}},\phi_{M,p_{d}},\phi_{N,p_{a}},\phi_{N,p_{d}}\right)}^{T}.$ Here, $\phi_{i,p_{j}}$ with *i*  =  M, N and *j*  =  *a*, *d* represents the |*p_j_* 〉 component on Site *i*, the lattice constant is taken as 1, *k* denotes the wave number along *x* direction, and † indicates the complex conjugate transpose. Note that in the formulations of tight‐binding method, the spatial and vector features of the *p*‐orbitals are smeared out and we only use |*p_a_* 〉 and |*p_d_* 〉 (instead of the full notations of |*p_a_* 〉, |*p_d_* 〉, **v**_*a*_ and **v**_*d*_) to represent the two orbitals.

**Figure 2:**
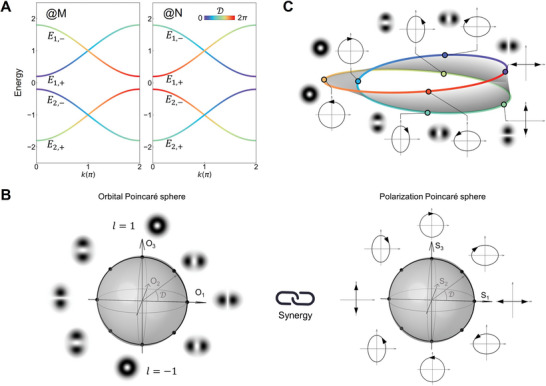
The synergy between SAM and OAM. A) Energy bands of the orbital lattice, incorporating D. M and N denote different sites. One cyclic evolution is illustrated by tracing D from 0 to 2π, exemplified for Site M in the *E*_1_ group and mapped onto B) the orbital and polarization Poincaré spheres. C) The cyclic evolution represented on a Möbius strip.

The phase difference between |*p_a_* 〉 and |*p_d_* 〉 components are obtained as $D=\arg(\frac{\phi_{i,p_{a}}}{\phi_{i,p_{d}}}).$ To track its evolution, we present the energy bands in **Figure** **2**A taking ϑ  = 60° , *t_t_* =  0, *t_l_* =   − 1 and *t_m_* =  0.4, where D is visualized through color‐coding (more details can be found in Section S2, Supporting Information). There are four bands grouped in two, denoted by *E*_1_ and *E*_2_ with + and − signs representing positive and negative group velocity, respectively. In the *E*_1_ group, D undergoes an evolution from 0 to 2π, with one half following the *E*_1,+_ band and the other following the *E*_1,−_ band. In the *E*_2_ group, the evolution is symmetric to the *E*_1_ group with respect to the zero‐energy. For different Sites M and N, D exhibits the opposite evolution. These observations elucidate the intricate dependence of D on *k*, which is intimately tied to the symmetries of the system (see Section S3, Supporting Information).

Associated with the evolution of D, the OAM and SAM exhibit a full cycle of synergy. To visualize the cyclic evolution, we employ the Poincaré sphere representations.^[^ ^32^ ^]^ Typically, a polarized state can be described on a sphere spanned by the Stokes parameters {*S*_1_,*S*_2_,*S*_3_}  =  {cos 2φcos 2χ, sin 2φcos 2χ, sin 2χ}, with 2φ and 2χ respectively the azimuthal and ellipticity angles. This way to represent polarization states has been generalized to also describe OAM states, known as the Poincaré sphere analog spanned by the orbital parameters {*O*_1_,*O*_2_,*O*_3_}.^[^ ^33^, ^34^ ^]^ It has the same mathematical formulations as the Poincaré sphere for polarization states, but delivers different physical meanings.^[^ ^33^ ^]^ In our scenario, the OAM and polarization states are superpositions of the orbital and orientation bases, yielding $|p_{d}\rangle +e^{iD}\left|p_{a}\right\rangle$ and $v_{d}+e^{iD}v_{a}$, respectively. This choice of eigenbases pins the evolution onto the *O*_1_‐*O*_3_ or *S*_1_‐*S*_3_ plane (corresponding to φ  =  0).

A full cycle of evolution can be tracked by D=2χ running over [0, 2π], as shown in Figure 2B, exemplified for Site M in the *E*_1_ group. Here, the OAM states are illustrated by the spatial mode profiles and the polarization states are presented using polarization ellipses for better visualizations (see Experimental Section for their calculations). Initially, at *E*_1,+_(*k*  =  0), the OAM state is expressed as |*p_d_* 〉 + |*p_a_* 〉, corresponding to a *p*‐orbital with orientation along the *x*‐direction. This state can be mapped to the east of the equator of the orbital Poincaré sphere, in synergy with a horizontal linear‐polarization **v**_*d*_ + **v**_*a*_ in the polarization Poincaré sphere. As *k* increases, the linear‐polarization transforms into elliptical‐polarizations, associated with distorted *p*‐orbitals. Upon reaching the poles, the evolution state becomes a perfect vortex and is simultaneously circularly‐polarized. For different sites, the evolution occurs in opposite directions, resulting in vortices with topological charges of *l* = ± 1 and circular‐polarizations with opposite handedness, i.e., |*p_d_* 〉 + *i*|*p_a_* 〉 and $\frac{\sqrt{2}}{2}e^{i\frac{\pi }{4}}{\left[\begin{array}{cc} 1 & i \end{array}\right]}^{T}$ for Site M at the north pole and |*p_d_* 〉 − *i*|*p_a_* 〉 and $\frac{\sqrt{2}}{2}e^{-i\frac{\pi }{4}}{\left[\begin{array}{cc} 1 & -i \end{array}\right]}^{T}$ for Site N at the south pole (note that the circular‐polarizations carry additional phase factors compared to the conventions; this is attributed to our choice of eigenbases and more discussions can be found in Section S4, Supporting Information). Continuous increase in *k* leads to the evolution from the north pole to the west of the equator for Site M and from the south pole to the west of the equator for Site N. Correspondingly, the OAM state returns to the *p*‐orbital and the polarization to the linear‐polarization, only with a vertical orientation. This completes half of the evolution. By following the *E*_1,−_ band, the other half unfolds, precisely reversing the first half of M‐evolution for Site N and the first half of N‐evolution for Site M. In this way, a full cycle of SAM‐OAM synergy is realized. For the *E*_2_ group, a similar analysis can be performed.

It is known that a cyclic evolution on a Poincaré sphere generates a nontrivial Pancharatnam‐Berry geometric phase θ_*g*_ =  Ω/2, where Ω is the solid angle enclosed by the cyclic evolution.^[^ ^35^, ^36^ ^]^ In our case, the cyclic evolution encloses half of the Poincaré sphere, giving rise to θ_*g*_ =  π. Such nonzero geometrical phase in the momentum space indicates nontrivial topology of the periodic lattice (see Section S5, Supporting Information). It also suggests that the eigenstates can be represented on a Möbius strip with 4π periodicity.^[^ ^37^, ^38^, ^39^ ^]^ This is consistent with our observation, i.e., the eigenstates return to their initial states only after 4π change of *k* (or 2π change of D). Figure 2C illustrates how one cycle of the SAM‐OAM synergy is represented on the Möbius strip. By precisely traversing the edge of the strip once, an *x*‐oriented *p*‐orbital mode (marked by the purple dot) evolves into a perfect vortex with *l* = +1 (the blue dot), then transforms into its *y*‐oriented counterpart (the green dot), landing on the opposite side to its initial position. Only traversing another perfect vortex with *l* = −1 (the orange dot), this mode returns to its initial position. Concurrently, the polarization undergoes a synchronized evolution, transitioning from a horizontal linear‐polarization to a right circular‐polarization, followed by a vertical linear‐polarization, then transforming into a left circular‐polarization, and finally returning to the initial horizontal‐polarization. Notably, each pair of eigenstates located at the opposite sides of the Möbius strip corresponds to a pair of states in opposite Poincaré hemispheres (e.g., east to west or north to south).

Simultaneously manipulating anisotropic orbitals and their orientations opens up opportunities to synchronize OAM and SAM, uncovering a fundamental connection between these angular momenta. We argue that this principle is universal and can be extended to a general selection of orbitals, orientations, and physical systems, offering fresh perspectives and versatile methods for angular momentum manipulations (see more discussions in Section S6, Supporting Information).

### 2.3 Experimental Demonstration

**Figure 3:**
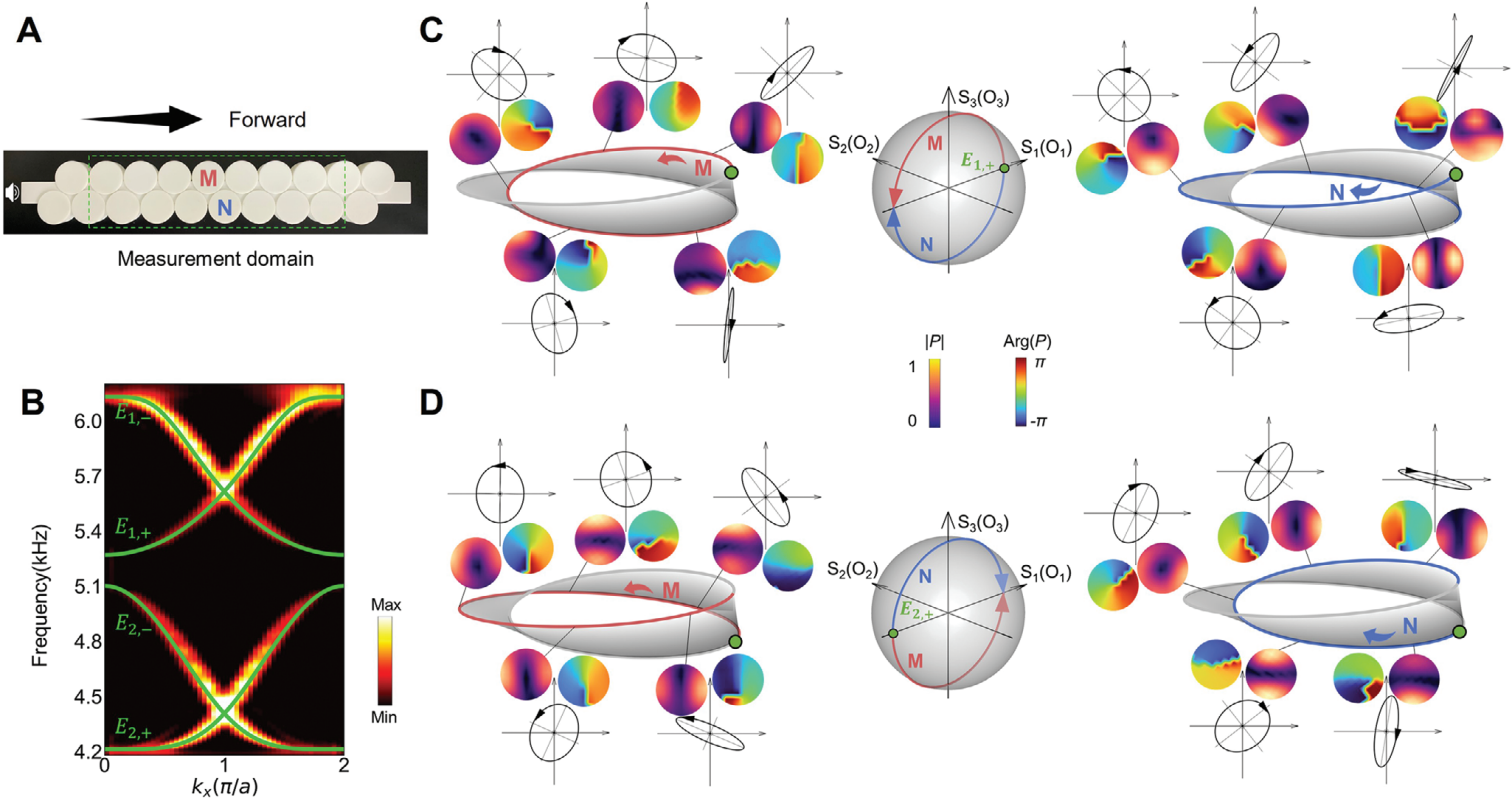
Experimental demonstration of the SAM‐OAM synergy. A) A photo of the fabricated acoustic lattice. B) Numerically calculated (green curves) and experimentally measured (color map) band structures for the acoustic lattice. C) Measured acoustic pressure fields |*P*|, their phase distributions Arg(*P*), and polarization ellipses of velocity fields for cavities M and N following *E*_1,+_ evolution. Both representations on the Möbius strip and indication on the polarization (orbital) Poincaré sphere are provided (the Poincaré sphere representations of the experimental data can be found in Section S7, Supporting Information). D) The same as (C), only for *E*_2,+_ evolution.

We fabricate the acoustic lattice using 3D‐printing, as shown in **Figure** **3**A. The band structures are presented in Figure 3B, both numerically calculated and experimentally measured (see details in Methods), which exhibit a high agreement with the energy bands in Figure 2A, despite a slight breaking of chiral symmetry. This arises from the relatively wide coupling tube compared to the acoustic cavities, which is intentionally designed to facilitate experimental implementation for a broad frequency bandwidth and reduced scattering during sound wave propagation. This design inevitably introduces long‐range couplings and a resonant frequency shift, leading to the breaking of chiral symmetry. In Section S8 (Supporting Information), we fit the tight‐binding model to account for these effects and find that, importantly, despite the breaking of chiral symmetry, the SAM‐OAM evolution and their synergized relationship remain unaffected. To demonstrate, we present the cyclic evolution and SAM‐OAM synergy along the *E*_1,+_ band in Figure 3C. The pressure fields and their phase distributions are measured as spatial OAM features and the polarization ellipses are depicted based on velocity field measurements, representing vector polarization features. Note that only the evolution along the *E*_1,+_ band (with increased frequency) is measured, with the red arrow indicating M‐evolution and the blue arrow N‐evolution. As discussed earlier, the evolutions on Sites M and N have opposite directions and therefore such measurements complete a full cycle as indicated by the evolution paths on the polarization or orbital Poincaré sphere. Tracking the M‐evolution, it is observed to precisely agree with the theoretical prediction, i.e., from the east of the equator passing the north pole to the west of the equator, an *x*‐oriented *p*‐orbital (accompanying the horizontal linear‐polarization) transforms into a vortex with *l* = +1 (accompanying the right‐circular‐polarization), then returns to a *y*‐oriented *p*‐orbital (accompanying the vertical linear‐polarization). For the N‐evolution, it is also from an *x*‐oriented *p*‐orbital to a *y*‐oriented *p*‐orbital, only passing a vortex with *l* = −1 (at the south pole).

Similar to Figure 3C, Figure 3D presents the cyclic evolution along the *E*_2,+_ band. Again, both M‐ and N‐evolutions are measured, which exhibit consistent behaviors with the theory. In this case, the starting point is the west of the equator and the ending point is the east of the equator, with M‐evolution passing the south pole while the N‐evolution passing the north pole. Equivalently, these two evolutions also form a complete cycle. We point out that due to the technical difficulties at the band edges (with zero group velocity), our measurements only cover the *k* regime close to [0,2π]. Nonetheless, the measurements evidently reflect the fundamental features of the cyclic evolution with high agreements with the theory (see Section S9, Supporting Information).

### 2.4 A Spin‐Orbital‐Hall Effect

**Figure 4:**
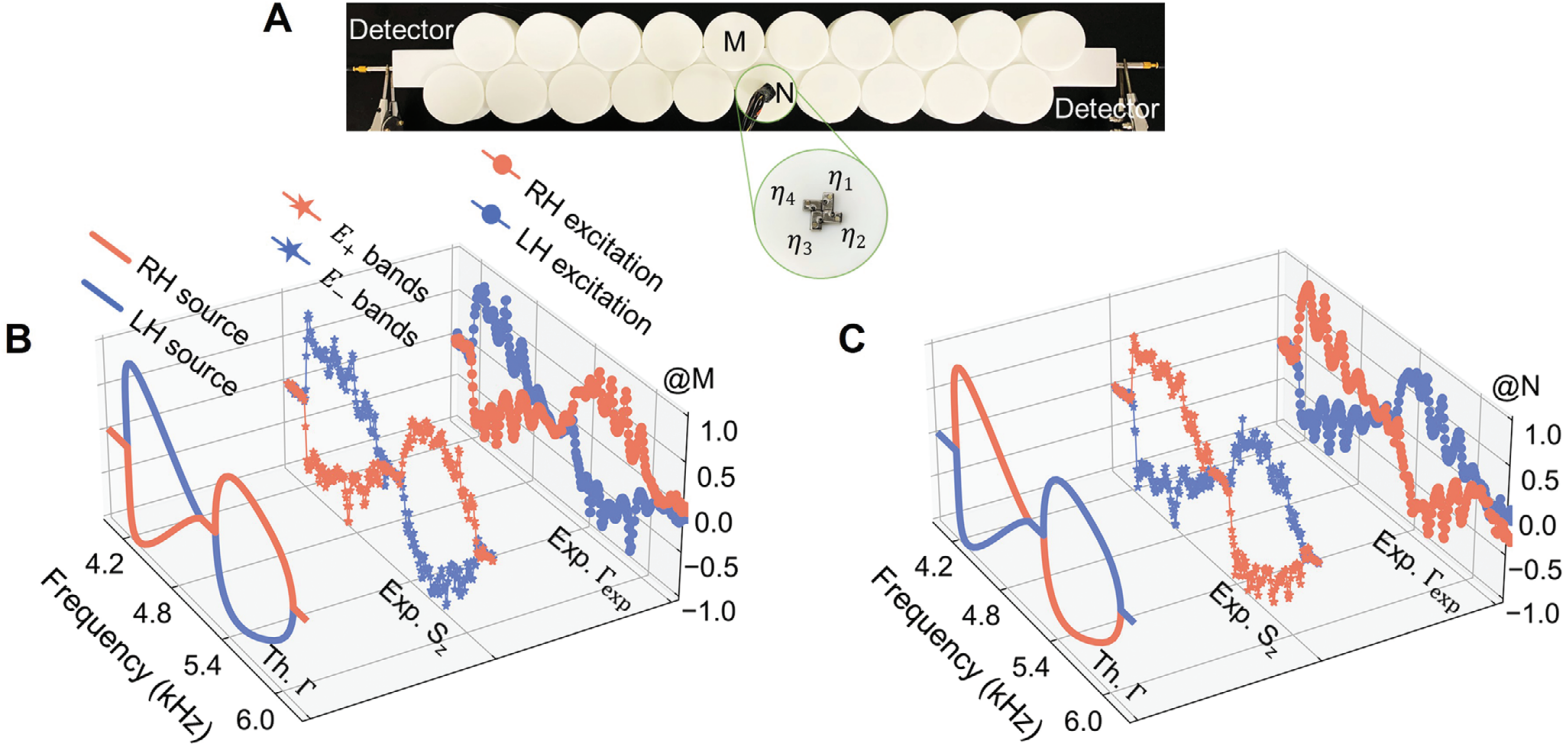
An acoustic spin‐orbital‐Hall effect. A) The experiment setup. B) Numerically calculated directional contrast Γ, and experimentally measured spin density *S_z_* and directional contrast Γ_exp_, for Site M. C) The same as (B), only for Site N.

As a fundamental property enabled by SOIs, SAM can be locked with the linear momentum, leading to a helicity‐dependent or handedness‐dependent wave propagation. This is known as the spin‐Hall effect.^[^ ^22^ ^]^ In our case, remarkably, SAM is synchronized with the OAM, promising a novel spin‐orbital‐Hall effect highlighting more interesting and intricate locking of handedness, directionality, spin density, and spatial mode profile. To probe the locking properties, we employ a chiral source generated by coupling four acoustic loudspeakers with a phase gradient (as shown in **Figure** **4**A, see Experimental Section for more details). This kind of source carries nonzero angular momentum that can couple with the SAM and correspondingly with the synchronized OAM, resulting in SAM‐OAM‐dependent sound transport.

Such dependence can be quantified by a directional contrast $\Gamma =\frac{\Gamma_{f}-\Gamma_{b}}{\Gamma_{f}+\Gamma_{b}}$, with Γ_*f*_ and Γ_*b*_ describing the likelihood of the wave propagation in the forward and backward directions, respectively. According to Fermi's golden rule,^[^ ^40^, ^41^, ^42^ ^]^ Γ_*f*_∝|s* · **V**_*f*_|^2^ and Γ_*b*_∝|s* · **V**_*b*_|^2^(see Section S10, Supporting Information). Here, $s=\frac{\sqrt{2}}{2}\;{\left[\begin{array}{cc} 1 & \pm i \end{array}\right]}^{T}$ denote the chiral sources carrying SAM with opposite handedness. **V**_*f*_ and **V**_*b*_ indicate the velocity fields for eigenstates with positive and negative group velocity, respectively. Physically, this determines how much of the energy in the source **s** is transferred into the polarization states defined by **V**_*f*_ and **V**_*b*_.

Figure 4B,C present Γ calculated based on the simulated band structures in Figure 3B, for Sites M and N, respectively. It is seen that a right‐handed chiral source with $s=\frac{\sqrt{2}}{2}\;{\left[\begin{array}{cc} 1 & i \end{array}\right]}^{T}$ (denoted by red lines) transfers energy into Site M (N) as a forward (backward) propagating state in the high‐frequency regime, but as a backward (forward) propagating state in the low‐frequency regime. When the source chirality is reversed, with $s=\frac{\sqrt{2}}{2}\;{\left[\begin{array}{cc} 1 & -i \end{array}\right]}^{T}$ (blue lines), the sign of Γ flips. This suggests that the right‐ (left‐) handedness couples to the polarization states with a preference in the north (south) hemisphere, which is reasonable given the polarization features analyzed in the cyclic evolutions.

Enabled by the cyclic evolution, more interestingly, the polarization‐dependent wave propagation is tunable with respect to different frequencies. Tracking the frequency axis in both Figure 4B,C, we see that the Γ‐curves display two maxima ≈4.4 and 5.6 kHz, precisely corresponding to the poles accommodating circular‐polarizations. At these points, the source energy is maximally transferred into a single mode, leading to unidirectional sound propagation. Away from the maxima, the source energy is split, with a large portion transferred into the preferable direction and a small portion into the opposite direction with reversed handedness. The transition ratio is quantified by Γ. In fact, such tunable polarization‐splitting is related to the change of spin density during the cyclic evolution. The spin density is defined as the angular momentum carried by the velocity fields, yielding S=ρ2ωIm(V∗×V).^[^ ^14^, ^15^ ^]^ It characterizes the acoustic polarizations of local velocity field rotations, with normalized **S** being zero representing linear‐polarizations, unity representing circular‐polarizations (positive for the right‐circular‐polarization and negative for the left‐circular‐polarization) and in between representing elliptical‐polarizations. Using the experimental data measured in Figure 3, we calculate *S_z_* (*S_x_* = *S_y_*  =  0 due to zero *V_z_*). As presented in Figure 4B,C, *S_z_*‐curves are consistent with the Γ‐curves (with oscillations from the finite size effect).

Remember that in our system, the SAM and OAM are synchronized and synergized, suggesting a tunable OAM‐splitting, accompanying the tunable polarization‐splitting. For experimental demonstration, we place two detectors at the right and left ends of the sample in Figure 4A to measure the output pressure signals in the forward (*P_f_*) and backward (*P_b_*) directions, respectively. An experimental directional contrast is obtained as $\Gamma_{\exp}=\frac{|P_{f}\left|-\right|P_{b}|}{|P_{f}\left|+\right|P_{b}|}$ and plotted in Figure 4B,C. Again, Γ_exp_‐curves exhibit high agreements with the calculated Γ and measured *S_z_*, indicating the spatial OAM modes indeed exhibit a tunability in synergy with the polarization states (see more details in Section S11, Supporting Information).

## 3 Conclusion

We have demonstrated a full cycle of synergy between SAM and OAM by exploiting anisotropic *p*‐orbitals as eigenbases, whose spatial mode profiles and inherent orientations serve as ingredients to simultaneously manipulate OAM and SAM. The uncovering of such a unique and fundamental connection deepens our understanding of these angular momenta, which traditionally are considered independent and separate. It is especially prominent for scalar waves like acoustic waves, whose OAM is revealed to be naturally synchronized with the SAM due to the intrinsic connection between scalar pressure and vector velocity fields. The current model with simple acoustic cavities already glimpses into the rich dynamical physics of the SAM‐OAM synergy and tunable SAM‐OAM‐locked wave propagations. Facilitated with artificial designs, acoustic platforms offer unique opportunities to develop comprehensive models that can further incorporate diverse physical principles like topology, gauging, pumping, and non‐abelian physics. These advanced approaches hold great promise for versatile and sophisticated wave controls.^[^ ^43^, ^44^ ^]^ While investigating *p*‐orbitals in 1D, our principle can be applied to a more general selection of orbitals, orientations, spatial dimensions, and physical systems, with more degrees of freedom for rich SAM‐OAM interplay. In addition, synchronized SAM and OAM feature non‐separable states imprinting simultaneously spatial scalar and vector signatures with high potential in information and communications. Targeting the on‐chip information technologies, our principle may offer an integrated solution for coupled SAM‐OAM control with reduced fabrication complexity.

## 4 Experimental Section

### Acoustic Cavity, Calculations of its Resonant Modes and the Lattice Design

The designed acoustic cylindrical cavity had radius of *r*  =  2.16 cm and height of *h*  =  2.75 cm. A series of resonant modes within this cavity were identified, as depicted in Figure S1 (Supporting Information). These modes are numerically calculated using the commercial finite‐element simulation software COMSOL MULTIPHYSICS, by applying the 3D acoustic module and conducting the eigenfrequency evaluations. The mass density and sound velocity of air are taken as 1.21 kg m^−3^ and 343  m s^−1^, respectively. The outer boundaries of the cavity were set as hard boundaries.

Through periodic modulation and directional coupling, an acoustic lattice to investigate the SAM‐OAM synergy was designed, as shown in Figures 3A, 4A and Figure S1. The distance between the centers of cavities M and N is set as *d*  =  5 cm and the dislocation angle is ϑ  =  60°. Thus, the period of the lattice was *a*  =  2*d* cos ϑ =  5 cm. The acoustic cavities were coupled via a rectangular air channel with width and height taken as *W*  =  2.6 cm and *L*  =  2.16 cm, respectively.

### Band Structure Calculations and Measurements

For the acoustic lattice shown in Figure 3A, we numerically calculate its band structures, depicted in Figure 3B using green curves. The calculations are conducted again using COMSOL, by applying the 3D acoustic module and conducting the eigenfrequency evaluations. One unit cell is considered, with the left and right boundaries (along the *x*‐direction) set as periodic.

Experimentally, the sample by 3D printing was fabricated, using photosensitive resin as an acoustically rigid material. The wall thickness (of both the cavities and the air channel) was taken as 3 mm. To measure the band structures, a lattice sample with length of 10 unit cells was prepared. An acoustic loudspeaker (HiVi B1S) was placed at one end of the rectangular air channel to generate an acoustic signal, which was guided into the sample to excite forward (backward) propagating waves when the loudspeaker was placed at the left (right) end of the air channel. An acoustic detector (GRAS‐46BE ¼‐inch microphone) was used to probe the excited pressure field at the center of each cavity. The data were collected using a DAQ card (NI 9234). The obtained real‐space data are further taken for the Fourier transform using Matlab's built‐in *fft* function to get the band structures in momentum space, where the zeros padding method was applied to increase resolution. The measured band structures are shown in Figure 3B using a color map.

### Cyclic Evolution Measurements

Measurements of the cyclic evolutions include both the spatial pressure field distributions and the vector velocity field characteristics, using the same sample as that for band structure measurements. Upon a fixed excitation frequency, the pressure fields and their phase distributions are measured by step‐by‐step scanning two neighboring cavities (corresponding to Sites M and N) in the middle of the finite sample, with a step of 5 mm. The data were collected and processed to generate the color maps in Figure 3C,D. The excitation frequencies for the high‐frequency band *E*_1,+_ evolution were 5.31, 5.5, 5.65, 5.85, and 6.12 kHz, and for the low‐frequency band *E*_2,+_ evolution, they were 4.22, 4.27, 4.38, 4.7, and 5.05 kHz.

For the velocity field measurements, a similar procedure was adopted, i.e., an acoustic loudspeaker was placed at one end of the lattice sample and the velocity field was probed at the two neighboring cavities in the middle of the sample. Only now, instead of common acoustic detectors that could only measure the scalar pressure fields, *an acoustic vector sensor* (¼‐inch AVS‐2D) was utilized, which can directly measure the vector velocity fields,^[^ ^12^, ^16^ ^]^ yielding two perpendicular components $V_{x}=V_{0x}\;e^{i\zeta_{x}}$ and $V_{y}=V_{0y}\;e^{i\zeta_{y}}$ with *V*_0_ and ζ representing their amplitudes and phases, respectively.

To calculate the polarization ellipses, the imaginary parts for clarity and normalize the velocity components was omited, leading to

(1) $$V_{x}\;\left(t\right)=V_{0x}^{\prime }\;\cos\left(\omega t+\zeta_{x}\right)$$

(2) $$V_{y}\;\left(t\right)=V_{0y}^{\prime }\;\cos\left(\omega t+\zeta_{y}\right)$$

with

(3) $$V_{0x}^{\prime }=\frac{V_{0x}}{\sqrt{V_{0x}^{2}+V_{0y}^{2}}}$$

(4) $$V_{0y}^{\prime }=\frac{V_{0y}}{\sqrt{V_{0x}^{2}+V_{0y}^{2}}}$$

Here, the acoustic waves are time‐harmonic was assumed. With simple algebra, it is seen that as time varies, the endpoint of the vector velocity field moves on an ellipse yielding

(5) $$\frac{V_{x}^{2}}{{\left(V_{0x}^{\prime }\right)}^{2}}+\frac{V_{y}^{2}}{{\left(V_{0y}^{\prime }\right)}^{2}}-2\frac{V_{x}V_{y}}{V_{0x}^{\prime }V_{0y}^{\prime }}\;\cos\Delta \zeta =\sin^{2}\Delta \zeta$$

where Δζ  = ζ_*y*_ − ζ_*x*_ is the phase difference between *V_x_* and *V_y_* components. The velocity field is right‐handed polarization when 0 < Δζ < π and left‐handed polarization when π < Δζ < 2π. The polarization ellipses are correspondingly generated by tracing ω*t* from [0,  2π]. It is pointed out that instead of step‐by‐step scanning, for the velocity fields, several spatial locations were only measured. Nonetheless, the velocity fields near the central location exhibit almost consistent polarization characteristics (see Section S7 and Movies S1 and S2, Supporting Information). For clarity, in Figure 3C,D in the main text, we only present the measurements at the center of the acoustic cavities.

In the theoretical analyses, we apply a similar procedure to calculate the polarization ellipses as shown in Figure 2B,C, using the vector bases $v_{d}=\frac{\sqrt{2}}{2}{\left[\begin{array}{cc} 1 & -1 \end{array}\right]}^{T}$ and $\;v_{a}=\frac{\sqrt{2}}{2}\;{\left[\begin{array}{cc} 1 & 1 \end{array}\right]}^{T}$. Note that there is reasonable discrepancy between the theoretical calculations (Figure 2C) and the experimental measurements (Figure 3C,D). There are several reasons that may lead to such discrepancy. First, the acoustic lattice design uses a relatively wide air channel to couple the cavities, which may induce long‐range coupling and lead to deviations from the theoretical model. Second, in the experiments, a lattice sample with finite size is fabricated, which may generate scattering also responsible for the distortion. Third, in the measurements, we insert the acoustic vector sensor into the cavity, which can also sabotage the local velocity field distributions. Nonetheless, our measurements satisfyingly and evidently demonstrate the cyclic evolution, consistent with the theoretical prediction.

### Directional Contrast and Spin Density

The theoretical directional contrast Γ was calculated by extracting the eigen velocity fields at the center of cavities M and N from the numerically calculated band structures. Each time‐reversed pair of eigenstates yields a data set of **V**_*f*_ and **V**_*b*_, corresponding to the eigenstates with positive and negative group velocity, respectively. Then, apply Fermi's golden rule to determine how much of the chiral source energy can be transferred into the forward or backward direction.

In experimental measurements, four point‐sources with gradient phase modulation to mimic the chiral sources (see illustrations in Figure 4A) was injected, with η_1_ =  0, $\eta_{2}=\frac{3\pi }{2}$, η_3_ =  π and $\eta_{4}=\frac{\pi }{2}$ producing the chiral source with right‐handedness and $\eta_{1}=0,\;\eta_{2}=\frac{\pi }{2},\;\;\eta_{3}=\pi$ and $\eta_{4}=\frac{3\pi }{2}$ producing the chiral source with left‐handedness. Two detectors are placed at both ends of the sample to probe the forward and backward acoustic pressure signals *P_f_* and *P_b_*, which are further substituted into $\Gamma_{\exp}=\frac{|P_{f}\left|-\right|P_{b}|}{|P_{f}\left|+\right|P_{b}|}$ to obtain the experimental directional contrast Γ_exp_. For the measurements of Site M (Figure 4B, right panel), we place the chiral source in cavity M, and for the measurements of Site N (Figure 4C, right panel), the chiral source is placed in cavity N. The reason that we measure the pressure fields is for the purpose of demonstrating the SAM‐OAM synergy.

As further evidence, we also provide the spin density characterized by the vector velocity fields. This quantity is obtained from the cyclic evolution measurements that supply the data of **V**. These data are substituted into S=ρ2ωIm(V∗×V) to obtain the spin density **S**, whose result exhibits good agreement with both Γ and Γ_exp_.

## Conflict of Interest

The authors declare no conflict of interest.

## Supporting information



Supporting Information

Supplementary Movie 1

Supplementary Movie 2

## Data Availability

The data that support the findings of this study are available from the corresponding author upon reasonable request.
