# Supplementary material for: Cyclic Evolution of Synergized Spin and Orbital Angular Momenta
Source: Adv Sci (Weinh). 2024 Nov 28;12(3):2409377. doi: 10.1002/advs.202409377 (PMC11744633; doi:10.1002/advs.202409377)
Supplement: Supplementary file 1 — Supporting Information [file ADVS-12-2409377-s001.docx]

Supporting Information

Cyclic Evolution of Synergized Spin and Orbital Angular Momenta

Lei Liu^1^, Xiao-Chen Sun^1^, Yuan Tian^1^, Xiujuan Zhang^1^*, Ming-Hui Lu^1,2,3^* and Yan-Feng Chen^1,3^*

­­­

^1^National Laboratory of Solid State Microstructures and Department of Materials Science and Engineering, Nanjing University, Nanjing 210093, China.

^2^Jiangsu Key Laboratory of Artificial Functional Materials, Nanjing 210093, China.

^3^Collaborative Innovation Center of Advanced Microstructures, Nanjing University, Nanjing 210093, China.

*Corresponding authors: Xiujuan Zhang, xiujuanzhang@nju.edu.cn; Ming-Hui Lu, luminghui@nju.edu.cn; Yan-Feng Chen, yfchen@nju.edu.cn;

1. **Resonance spectrum of acoustic cylindrical cavity**

**
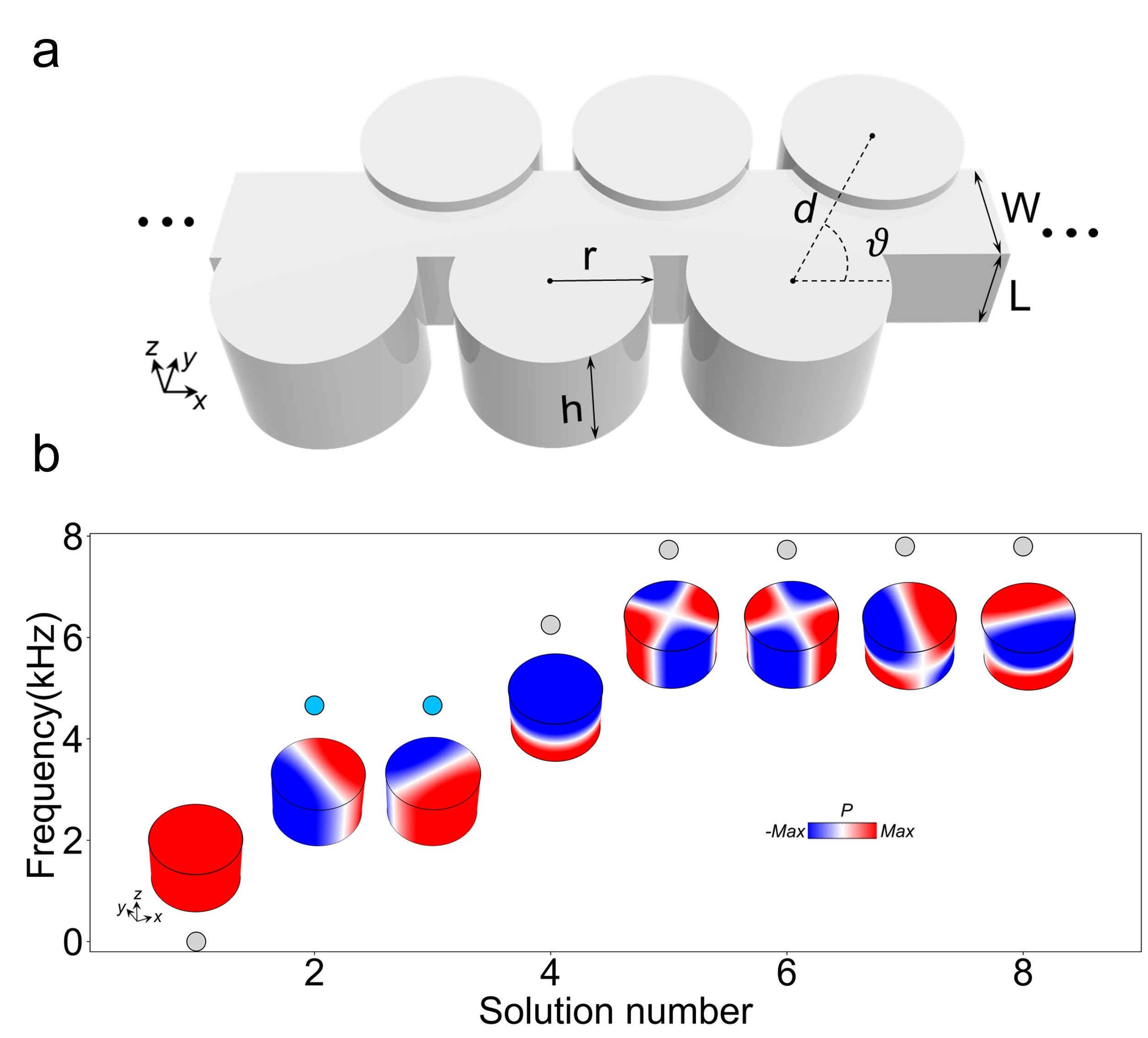
**

Figure S1. **a**, Schematics of the quasi-1D acoustic lattice. **b**, Resonant mode solutions in the acoustic cylindrical cavity in Figure 1A. The first- and second-order monopole resonances are observed at frequencies of 0 and 6.25 kHz, respectively; The resonant frequencies of degenerated first- and second-order dipole resonances are 4.66 and 7.8 kHz, respectively; The degenerated first-order quadrupole resonances are located at 7.73 kHz.

We calculated the resonance spectrum of the acoustic cylindrical cavity depicted in Figure S1a, as shown in Figure S1b. The first- and second-order monopole resonances are observed at 0 and 6.25 kHz, respectively. The resonant frequencies of the degenerate first- and second-order dipole resonances are 4.66 and 7.8 kHz, respectively, and the degenerate first-order quadrupole resonances are located at 7.73 kHz. In the main text, we use two degenerate and orthogonal first-order dipole resonances to mimic orthogonal $p$-orbitals $|\left. p_{a} \right\rangle$ and $|\left. p_{d} \right\rangle$.

1. $\mathfrak{D}$ **expression and coupling coefficients.**

**2.1** $\mathfrak{D}$ **expression.**

The model is based on anisotropic $p$-orbitals. Thus, the transverse coupling is typically small and for simplicity, we set it as $t_{t}=0$ (see Supplementary Sections 2.2 and 2.3). Solving the eigenequation $H\left| \left. \psi\right\rangle=E \right|\left. \psi\right\rangle$ yields the following eigenenergies and eigenfunctions

$E_{1,+}=-t_{l}-2t_{m}\cos\frac{k}{2}, {|\left. \psi\right\rangle}_{1,+}=\frac{1}{2}\left[ \begin{matrix} \begin{matrix} -e^{\frac{ik}{2}} & -e^{ik} \end{matrix} & \begin{matrix} e^{\frac{ik}{2}} & 1 \end{matrix} \end{matrix} \right]^{T}$, (S1a)

$E_{1,-}=-t_{l}+2t_{m}\cos\frac{k}{2}, {|\left. \psi\right\rangle}_{1,-}=\frac{1}{2}\left[ \begin{matrix} \begin{matrix} e^{\frac{ik}{2}} & -e^{ik} \end{matrix} & \begin{matrix} {-e}^{\frac{ik}{2}} & 1 \end{matrix} \end{matrix} \right]^{T}$, (S1b)

$E_{2,+}=t_{l}-2t_{m}\cos\frac{k}{2}, {|\left. \psi\right\rangle}_{2,+}=\frac{1}{2}\left[ \begin{matrix} \begin{matrix} -e^{\frac{ik}{2}} & e^{ik} \end{matrix} & \begin{matrix} -e^{\frac{ik}{2}} & 1 \end{matrix} \end{matrix} \right]^{T}$, (S1c)

$E_{2,-}=t_{l}+2t_{m}\cos\frac{k}{2}, {|\left. \psi\right\rangle}_{2,-}=\frac{1}{2}\left[ \begin{matrix} \begin{matrix} e^{\frac{ik}{2}} & e^{ik} \end{matrix} & \begin{matrix} e^{\frac{ik}{2}} & 1 \end{matrix} \end{matrix} \right]^{T}$. (S1d)

There are four energy bands in total, grouped in two (denoted by $E_{1}$ and $E_{2}$, with $+$ and $-$ signs representing positive and negative group velocity, respectively). In each group, the two bands degenerate at $k=\pi$. The associated eigenfunctions, as anticipated, indeed exhibit a phase difference between $|\left. p_{a} \right\rangle$ and $|\left. p_{d} \right\rangle$ components, which is quantified by $\mathfrak{D=}\arg\left( \frac{\phi_{{i,p}_{a}}}{\phi_{{i,p}_{d}}} \right)$, where $i=M,N$ denote different sites. Remarkably, we observe that $\mathfrak{D}$ solely depends on the wave vector $k$ and therefore is free of choice on the coupling coefficients.

**2.2 Cases with** $\boldsymbol{t}_{\boldsymbol{t}}\boldsymbol{\neq0}$**.**


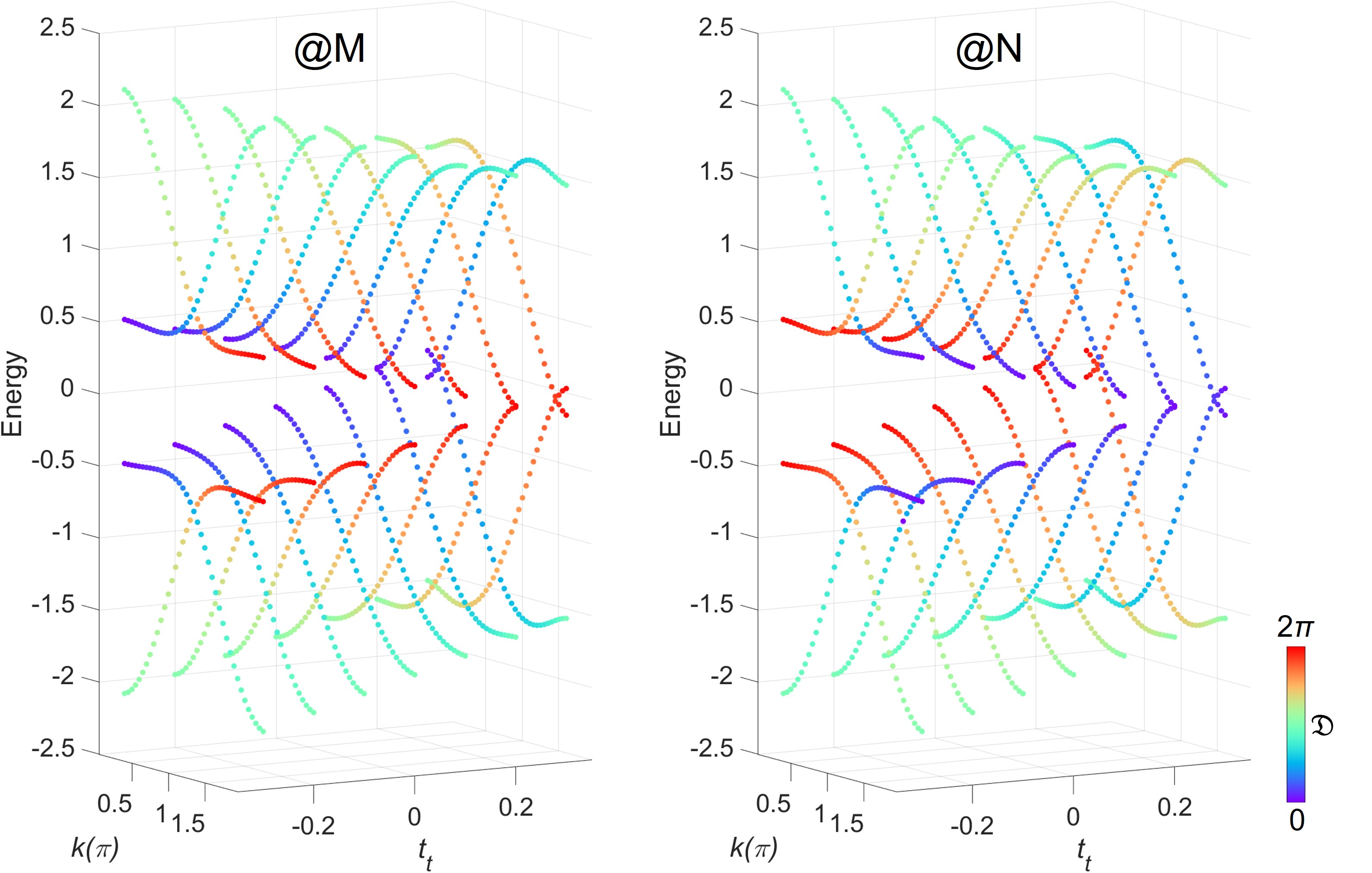


Figure S2. Band structures for Sites M and N with $t_{t}$ assigned values ranging from $-0.3$ to $0.3$, while keeping other parameters consistent with those discussed in the main text.

To provide a comprehensive discussion, we now consider cases where $t_{t}\neq0$. Given $\left| t_{t} \right|\ll\left| t_{m} \right|<\left| t_{l} \right|$, we take $t_{t}$ ranging from $-0.3$ to $0.3$. The corresponding band structures are shown in Figure S2. Despite minor distortions in the band shape and the diminishing bandgap with increasing $t_{t}$, the evolutions of $\mathfrak{D}$ align with those in the main text, completing a full range $\left[ 0, 2\pi\right]$. Furthermore, we calculate the amplitude ratio between two orbitals, quantified by

$\mathcal{A=}\left| \frac{\phi_{{i,p}_{a}}}{\phi_{{i,p}_{d}}} \right|$, (S2)

where $i=M,N$ denote different sites. Upon incorporating the numerical values into Equation (S2), it is observed that in each case, $\mathcal{A}$ equals 1. This suggests that the orbital and polarization states are consistent with those described in the main text, namely $\frac{\sqrt{2}}{2}\left( \left| \left. p_{d} \right\rangle+e^{i\mathfrak{D}} \right|\left. p_{a} \right\rangle\right)$ and $\frac{\sqrt{2}}{2}\left( \boldsymbol{v}_{d}+e^{i\mathfrak{D}}\boldsymbol{v}_{a} \right)$. Therefore, the value of the coupling coefficients $t_{t}$ does not influence the evolution of orbital and polarization states on the orbital and polarization Poincaré spheres.

**2.3 Relation between coupling coefficients and angle** $\boldsymbol{\vartheta}$**.**


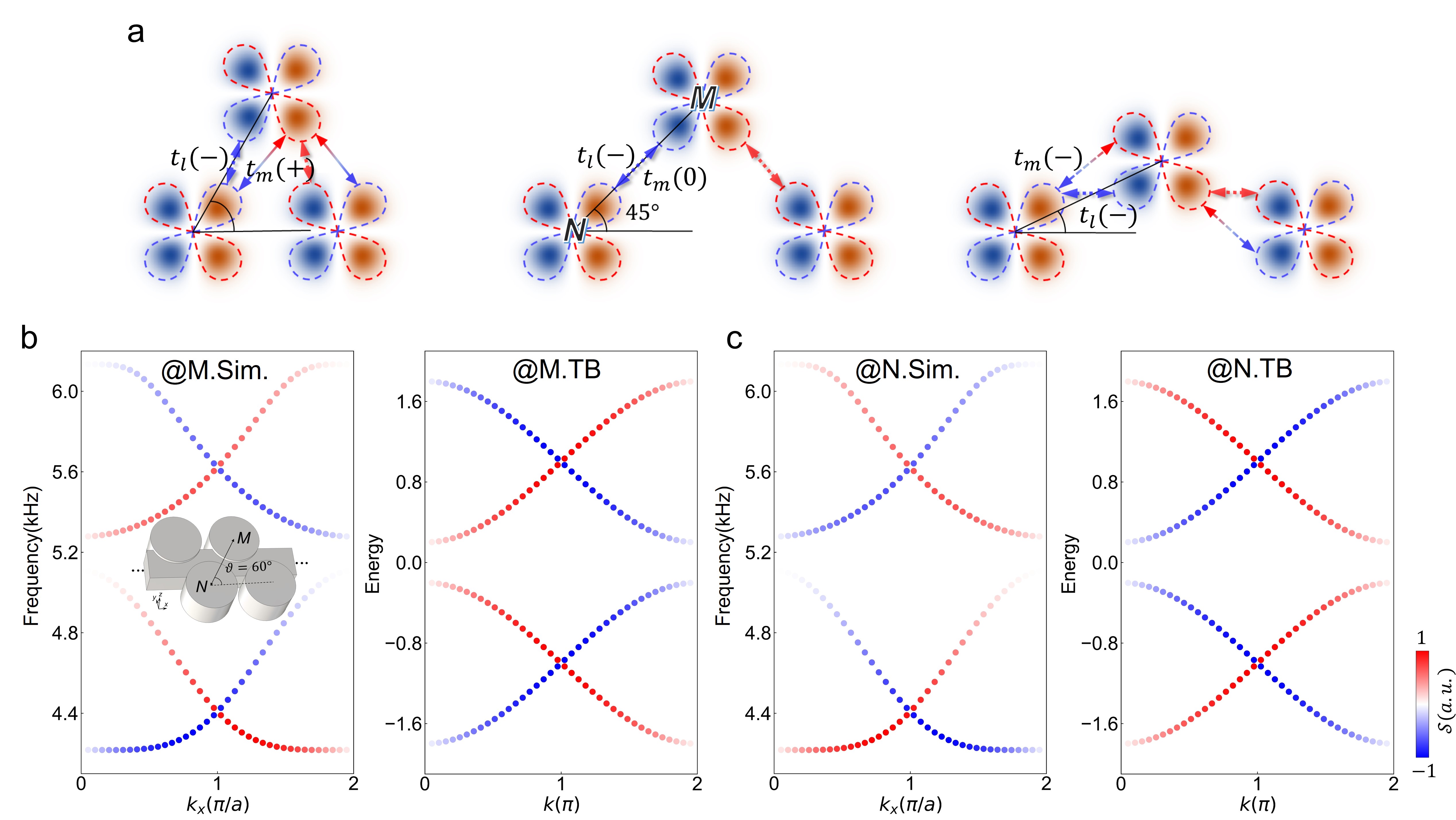


Figure S3. **a**, Sites M and N are dislocated at angle $\vartheta>{45}^{^{\circ}}$ (left plane), $\vartheta={45}^{^{\circ}}$ (middle plane), $\vartheta<{45}^{^{\circ}}$(right plane). **b**, Simulation band results with $\vartheta={60}^{^{\circ}}$and tight binding results with $t_{t}=0$, $t_{l}=-1$ and $t_{m}=0.4$ for Cavity and Site M. **c**, The same as **b**, only for Cavity and Site N.

Given that our model relies on anisotropic $p$-orbitals, their coupling coefficients are contingent on the overlap integral of their wavefunctions,^[1,2]^ which is associated with the angle $\vartheta$, as shown in Figure S3a. The coupling between $p_{a}$- and $p_{d}$-orbitals at the same site is always exactly zero due to the orthogonality of their wave functions. Additionally, the overlap integral between $p_{a}$- and $p_{d}$-orbitals at nearest sites, namely the crossed coupling $t_{m}$, is also zero when $\vartheta$ is equal to ${45}^{^{\circ}}$, as shown in the middle plane of Figure S3a. However, the crossed coupling becomes nonzero as $\vartheta$ deviates from ${45}^{^{\circ}}$. For ${45}^{^{\circ}}<\vartheta<{90}^{^{\circ}}$, as shown in the left plane of Figure S3a, the crossed coupling primarily involves the overlap integral between lobes of different $p$-orbitals carrying the same signs, resulting in a positive value of $t_{m}$. Conversely, for $0<\vartheta<{45}^{^{\circ}}$, as shown in the right plane Figure S3a, it mainly involves the overlap integral between lobes of different $p$-orbitals carrying opposite signs, leading to a negative value of $t_{m}$. Moreover, for each $\vartheta$, the longitudinal coupling $t_{l}$ is consistently negative due to different signs of the nearest lobes between the same orbitals. Taking into account the spatial extension of $p$-orbitals, $\left| t_{t} \right|\ll\left| t_{m} \right|<\left| t_{l} \right|$ holds true. Without loss of generality, the acoustic lattice with $\vartheta={60}^{^{\circ}}$ is designed in the main text, as shown in the inset of Figure S3b. Thus, we set $t_{t}$, $t_{l}$ and $t_{m}$ as $0$, $-1$ and $0.4$, respectively.

The phase difference $\mathfrak{D}$ between two vector velocity bases $\mathbf{v}_{a}$ and$\mathbf{v}_{d}$leads to nonzero SAM. In the TB model, the SAM of polarization states$\mathbf{v}=\frac{\sqrt{2}}{2}\left( \mathbf{v}_{d}+e^{i\mathfrak{D}}\mathbf{v}_{a} \right)$ can be defined as^[3]^

$\mathcal{S}^{'}\propto Im\left( \mathbf{v}^{*}\times\mathbf{v} \right)$. (S3)

For the SAM in an acoustic lattice, it can be evaluated by integrating the SAM density in the vector velocity field $\mathbf{V}$ over the entire cavity, expressed as

$\mathcal{S\propto}∰ Im\left( \mathbf{V}^{*}\times\mathbf{V} \right)d\boldsymbol{r}$. (S4)

We calculate the band structures to validate the relation between coupling coefficients and angle $\theta$, incorporating the normalized SAM of eigenstates in the TB model and acoustic lattice for Sites or Cavities M and N, as shown in Figure S3b and c, respectively. The simulation results agree well with the TB results.

1. **Symmetries and their effects on the evolutions of Sites M and N.**


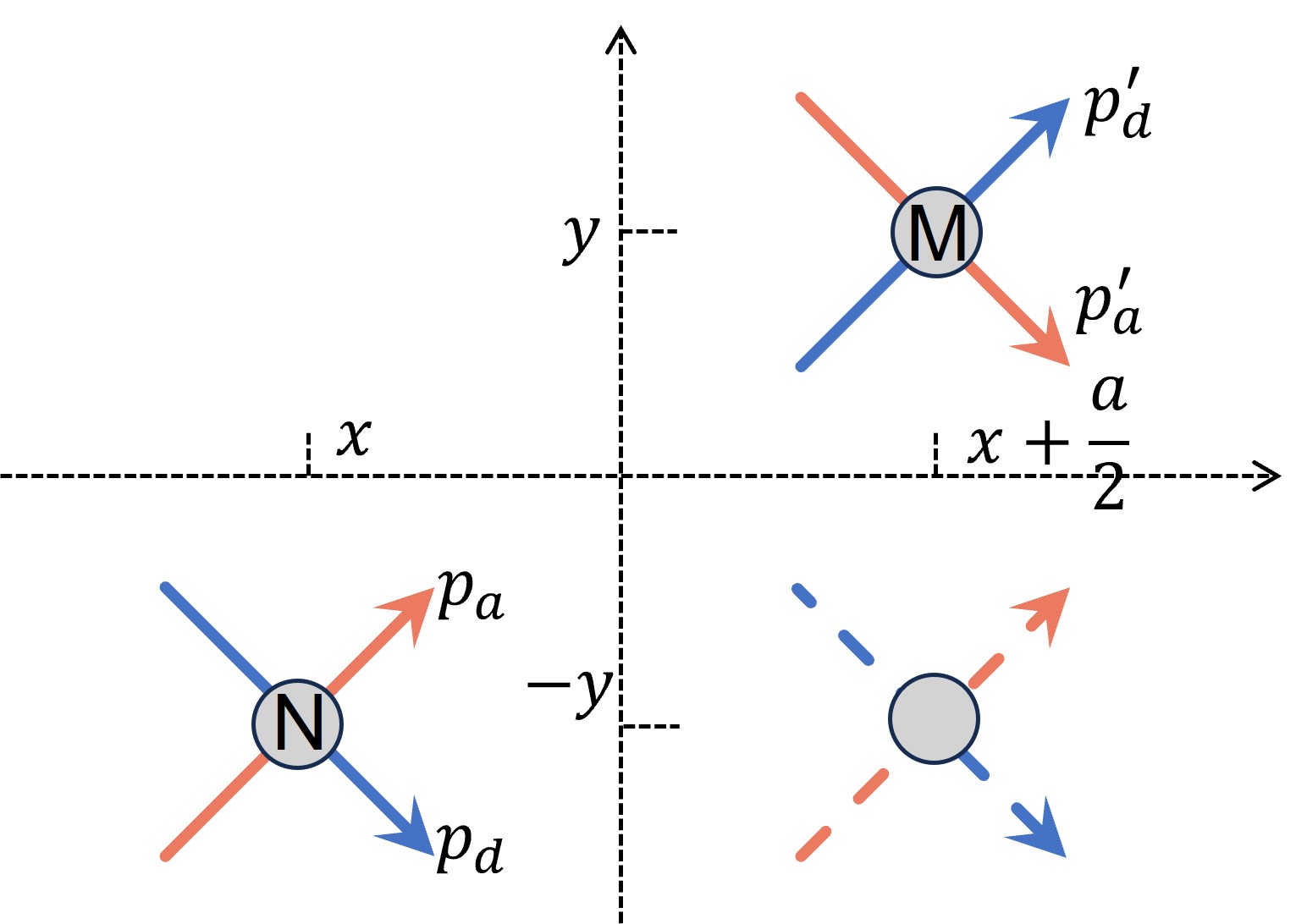


Figure S4. Glide symmetry $\mathcal{G}_{2}$ is composed of a translation symmetry $L_{x}: x\to x+\frac{1}{2}$ and a reflection symmetry $R_{y}: -y\to y$. This symmetry operation acts on the vector nature of $p$-orbitals, leading to opposite evolutions on Sites M and N.

The complex relationship between $\mathfrak{D}$ and $k$ is closely linked with the system's symmetries. Firstly, the chiral symmetry $\mathcal{C}H\left( k \right)\mathcal{C}^{-1}=-H\left( -k \right)$ with $\mathcal{C=}\sigma_{z}\bigotimes I_{2\times2}K$ (with $\sigma_{z}$ the Pauli matrix, $I$ the identity matrix and $K$ the complex conjugation) ensures the evolution of $\mathfrak{D}$ in $E_{1}$ and $E_{2}$ groups are symmetric with respect to the zero-energy due to $H\left( -k \right)\mathcal{C}\left| \left. \psi\right\rangle=-E\mathcal{C} \right|\left. \psi\right\rangle$.

Secondly, the system also obeys a non-symmorphic glide symmetry $\mathcal{G}_{2}:\left( x,y \right)\to\left( x+\frac{1}{2},-y \right)$, which is a combination of a translation symmetry $L_{x}: x\to x+\frac{1}{2}$ and a reflection symmetry $R_{y}: -y\to y$. This glide symmetry, when applied to two orthogonal $p$-orbitals, results in opposite evolutions for Sites M and N, as shown in Figure S4. The relationships for $|\left. \phi_{p_{a}} \right\rangle$ and $|\left. \phi_{p_{d}} \right\rangle$ on both sites are expressed as follows

$|\left. \phi_{{M,p}_{a}} \right\rangle=\mathcal{G}_{2}|\left. \phi_{{N,p}_{a}} \right\rangle\mathcal{=R(-}2\theta)|\left. \phi_{{N,p}_{a}} \right\rangle$, (S5a)

$|\left. \phi_{{M,p}_{d}} \right\rangle=\mathcal{G}_{2}|\left. \phi_{{N,p}_{d}} \right\rangle\mathcal{=R(-}2\theta^{'})|\left. \phi_{{N,p}_{d}} \right\rangle$. (S5b)

Here, $\mathcal{R}\left( -2\theta\right)$ are the two-dimensional rotation matrix with a rotation angle $-2\theta$, where $\theta\left( \theta^{'} \right)$ is the angle between orientation of orbitals $p_{a}\left( p_{d} \right)$ and reflector ($y=0)$. Consequently, we deduce that $\mathfrak{D}_{M}=\mathfrak{-D}_{N}$, following the definition $\mathfrak{D=}\arg\left( \frac{\phi_{{i,p}_{a}}}{\phi_{{i,p}_{d}}} \right)$. This implies that the evolution direction of the eigenstates on Site M is reversed on Site N, as detailed in Supplementary Section 9. Glide symmetry $\mathcal{G}_{2}$ similarly influences both orbitals and polarization bases. Taking the polarization bases as an example, the polarization states on Site M can be represented as

$|\left. \psi_{M} \right\rangle=|\left. \phi_{{M,p}_{d}} \right\rangle+|\left. \phi_{{M,p}_{a}} \right\rangle=\frac{\sqrt{2}}{2}\left( \left[ \begin{matrix} 1 \\ -1 \end{matrix} \right]+e^{i\mathfrak{D}_{M}}\left[ \begin{matrix} 1 \\ 1 \end{matrix} \right] \right)$

$=\mathcal{R}\left( -2\cdot-\frac{\pi}{4} \right)\left| \left. \phi_{{N,p}_{d}} \right\rangle\mathcal{+R}\left( -2\cdot\frac{\pi}{4} \right) \right|\left. \phi_{{N,p}_{a}} \right\rangle=\frac{\sqrt{2}}{2}\left( \left[ \begin{matrix} 1 \\ -1 \end{matrix} \right]+e^{-i\mathfrak{D}_{N}}\left[ \begin{matrix} 1 \\ 1 \end{matrix} \right] \right)$. (S6)

In comparison, the polarization states on Site N are given by

$|\left. \psi_{N} \right\rangle=|\left. \phi_{{N,p}_{d}} \right\rangle+|\left. \phi_{{N,p}_{a}} \right\rangle= \frac{\sqrt{2}}{2}\left( \left[ \begin{matrix} 1 \\ -1 \end{matrix} \right]+e^{i\mathfrak{D}_{N}}\left[ \begin{matrix} 1 \\ 1 \end{matrix} \right] \right)$. (S7)

Obviously, the evolution direction of states on Site M is the reverse of those on Site N.

Additionally, combined with time-reversal symmetry $T$, $\mathcal{G}_{2}$ allows an anti-unitary symmetry operator $\Theta_{2}=\mathcal{G}_{2}\cdot T$, which acts on the eigenfunctions $|\left. \psi\right\rangle$ at $k=\pi$ following $\Theta_{2}^{2}\left| \left. \psi\right\rangle=e^{-ik} \right|\left. \psi\right\rangle=-|\left. \psi\right\rangle$. This leads to a Kramers-like double degeneracy, and guarantees a full cycle of $\mathfrak{D}$, consistent with the observations in Figure 2A.

1. **Poincaré sphere representation based on diagonal and antidiagonal polarization bases**

In the conventional expressions, polarized states are described using a two-dimensional Jones vector, which is based on orthonormal horizontal and vertical polarization vector bases. These vectors are represented as ${\frac{\sqrt{2}}{2}\left[ \begin{matrix} 1 & 0 \end{matrix} \right]}^{T}$ and ${\frac{\sqrt{2}}{2}\left[ \begin{matrix} 0 & 1 \end{matrix} \right]}^{T}$, respectively. Therefore, the right- and left-handed circular polarizations are expressed as

$\frac{\sqrt{2}}{2}\left[ \begin{matrix} 1 \\ 0 \end{matrix} \right]+i\frac{\sqrt{2}}{2}\left[ \begin{matrix} 0 \\ 1 \end{matrix} \right]=\frac{\sqrt{2}}{2}\left[ \begin{matrix} 1 \\ i \end{matrix} \right]_{RH}$, (S8a)

$\frac{\sqrt{2}}{2}\left[ \begin{matrix} 1 \\ 0 \end{matrix} \right]-i\frac{\sqrt{2}}{2}\left[ \begin{matrix} 0 \\ 1 \end{matrix} \right]=\frac{\sqrt{2}}{2}\left[ \begin{matrix} 1 \\ -i \end{matrix} \right]_{LH}$. (S8b)

In our case, the eigenbases are the diagonal and antidiagonal polarization bases, which are represented as ${\frac{\sqrt{2}}{2}\left[ \begin{matrix} 1 & -1 \end{matrix} \right]}^{T}$ and ${\frac{\sqrt{2}}{2}\left[ \begin{matrix} 1 & 1 \end{matrix} \right]}^{T}$ respectively. Consequently, the right- and left-handed circular polarizations are

$\frac{\sqrt{2}}{2}\left[ \begin{matrix} 1 \\ -1 \end{matrix} \right]+i\frac{\sqrt{2}}{2}\left[ \begin{matrix} 1 \\ 1 \end{matrix} \right]=\frac{\sqrt{2}}{2}e^{i\frac{\pi}{4}}\left[ \begin{matrix} 1 \\ i \end{matrix} \right]_{RH}$, (S9a)

$\frac{\sqrt{2}}{2}\left[ \begin{matrix} 1 \\ -1 \end{matrix} \right]-i\frac{\sqrt{2}}{2}\left[ \begin{matrix} 1 \\ 1 \end{matrix} \right]=\frac{\sqrt{2}}{2}e^{-i\frac{\pi}{4}}\left[ \begin{matrix} 1 \\ -i \end{matrix} \right]_{LH}$. (S9b)

Compared to the conventional expressions, these versions include additional phase factors due to the rotation of the orthogonal bases. However, these phase factors do not alter the essential characteristics of the Jones vectors.

1. **Geometric phase and topological states**


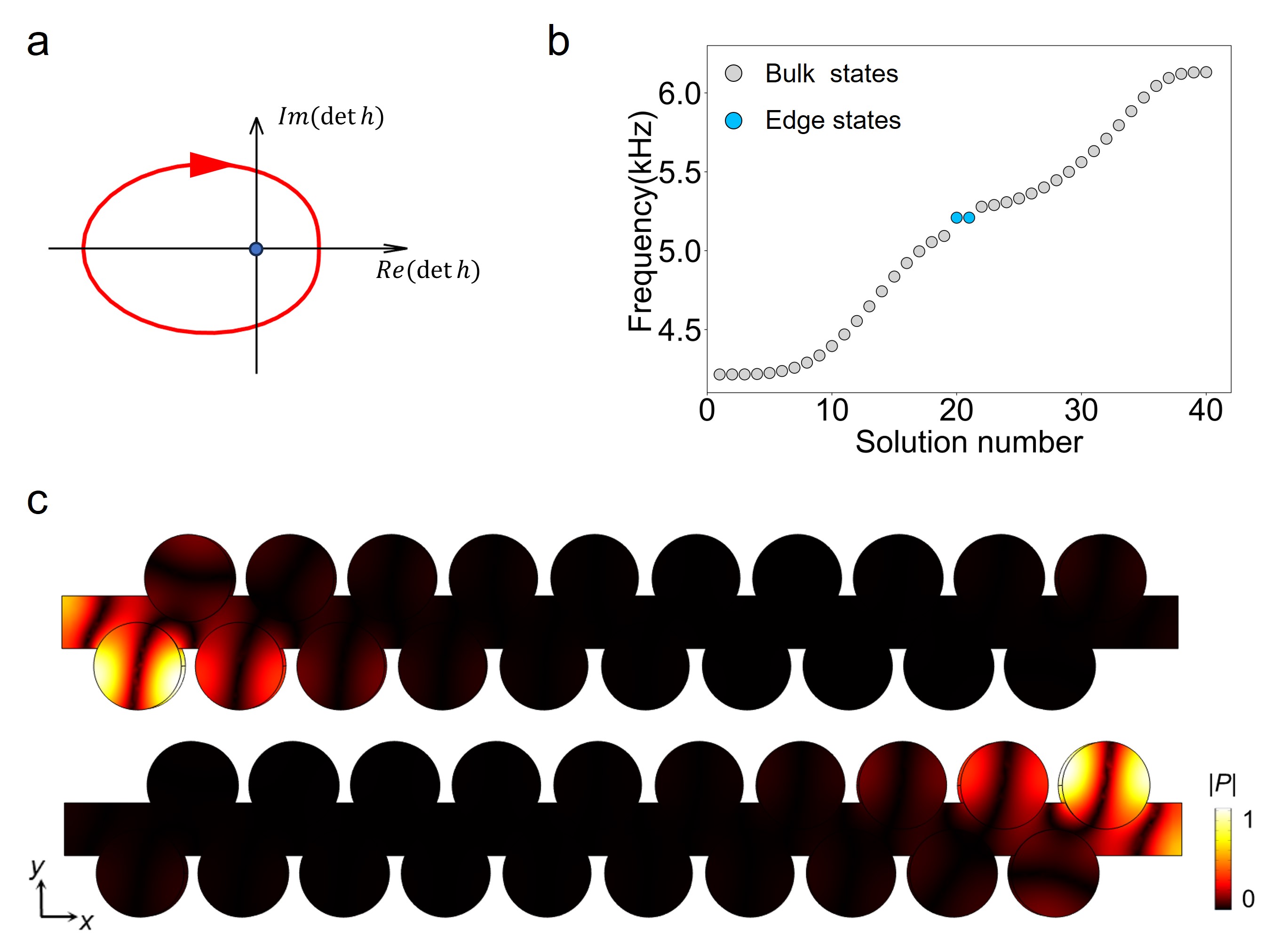


Figure S5. **a**, Illustration of the contour in the *com-plex* plane that $\det h$ (with parameters $t_{l}=-1$, $t_{m}=0.4$ and $t_{t}=0$) follows when $k$ varies from $0$ to $2\pi$. **b**, Eigenvalue spectrum for the structure in Figure 3A. **c**, Pressure field distributions of topological edge states.

According to the Berrey phase,^[4]^ the adiabatic evolution of the eigenstate in momentum space introduces an additional geometric phase. This phase is quantified by the Zak phase $Z$. The Hamiltonian of our system leads to the Zak phase being defined as^[5,6]^

$Z=\frac{i}{2}\int_{0}^{2\pi} dk\frac{d ln\left( det h \right)}{dk}=-\frac{1}{2}\oint d arg\left( det h \right)$. (S10)

The contour integral represents the path traced by $det h\left( k \right)$ as $k$ varies across the Brillouin zone. Under the conditions in the main text, namely $t_{l}=-1$, $t_{m}=0.4$ and $t_{t}=0$, the contour of $\det h$ encircles the zero point once, as shown in Figure S5a. This yields a Zak phase of $\pi$, which remarkably aligns with the geometric phase resulting from the eigenstates' evolution on the orbital and polarization Poincaré spheres. This non-trivial geometric phase suggests the existence of a pair of zero-energy eigenstates, or topological edge states. To validate this, we conducted simulations on a finite acoustic lattice comprising 10 unit cells, identical to the structure shown in Figure 3A. The eigenvalue spectrum is shown in Figure S5b, and reveals two degenerate edge states at the frequency of 5.21 kHz within the bandgap. Figure S5c displays the corresponding pressure field distributions of these topological edge states. The evolutions of OAM and SAM states on the orbital and polarization Poincaré spheres offer a method for directly measuring the evolution of eigenstates in momentum space. This approach enables the immediate observation of the topological characteristics in the system.

1. **Investigations into the universality of synergy between OAM and SAM.**

**6.1 Analytical display of the synergy between OAM and SAM.**


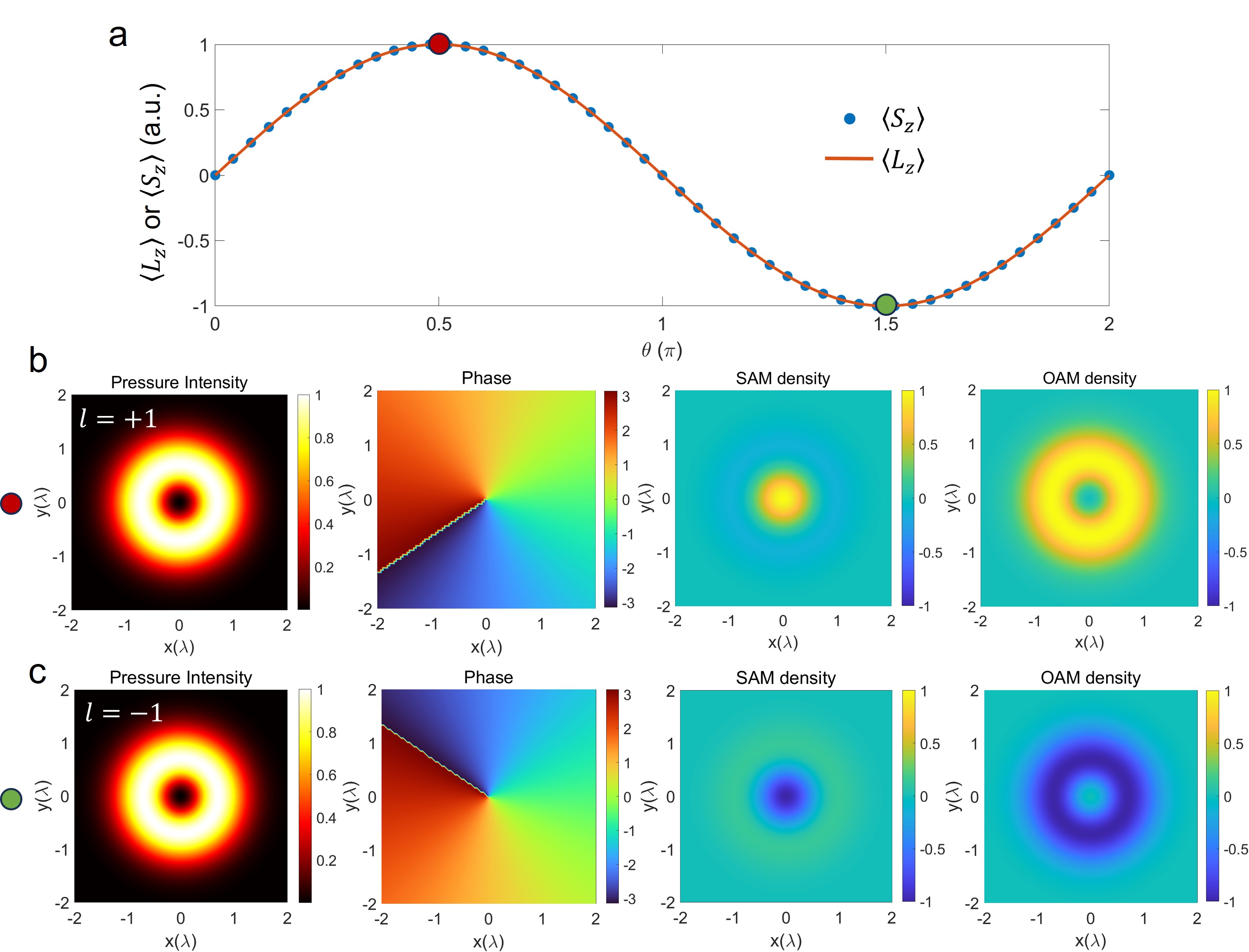


Figure S6. **a**, Normalized OAM $\left\langle L_{z} \right\rangle$ (orange line) and SAM $\left\langle S_{z} \right\rangle$ (blue dots) as functions of $\theta$. **b**, **c**, Acoustic pressure fields $\left| P \right|$, phase $arg\left( P \right)$, OAM and SAM density distributions for $\theta=\frac{\pi}{2}$(topological charge $l=1$) and $\frac{3\pi}{2}$ (topological charge $l=-1$), respectively. ($z=5\lambda$)

In Figure 1A and 1B of the main text, we show the vector velocity fields of *p* resonance are aligned with the orientations of scalar pressure fields. Such a correspondence is inherently rooted in the incompressible linear Euler equation

$\rho\frac{\partial\mathbf{V}}{\partial t}=-\nabla P$, (S11)

where $\rho$ represents the mass density, $\mathbf{V}$ denotes the velocity field and $P$ describes the pressure field. Assuming the acoustic waves are time-Harmonic, Equation (S11) can be re-written as

$\mathbf{V}=\frac{i}{\rho\omega}\nabla P$, (S12)

with $\omega$ being the angular frequency. Equation (S12) tells us the vector velocity field is connected with the gradient of the scalar pressure field, providing an ideal condition to implement the SAM-OAM synergy. In order to validate the universality of synergy between OAM and SAM, we investigate a Gaussian acoustic pressure filed, described by

$P\left( x,y \right)=P_{0}\left( x+e^{i\theta}y \right)e^{-\frac{x^{2}+y^{2}}{\lambda^{2}}}e^{ikz}$, (S13)

where $k$ ($=\frac{\omega}{\sqrt{\rho\beta}}$ with the bulk modulus $\beta$) is the wave number, and $P_{0}$ is given amplitude (initial pressure). The term $x+e^{i\theta}y$ induces the evolution of nontrivial OAM as $\theta$ varies. According to equation S12, the vector acoustic velocity field in the *x-y* plane is deduced as

$\mathbf{V}\left( V_{x},V_{y} \right)\boldsymbol{=}\frac{iP_{0}}{\rho\omega}\left[ 1-\frac{2x\left( x+e^{i\theta}y \right)}{\lambda^{2}}\boldsymbol{,}e^{i\theta}-\frac{2y\left( x+e^{i\theta}y \right)}{\lambda^{2}} \right]e^{ikz}e^{-\frac{x^{2}+y^{2}}{\lambda^{2}}}$. (S14)

We then evaluate the global characteristics of OAM and SAM by integrating the OAM and SAM density over the entire *x-y* plane. These are respectively expressed as^[15]^

$\left\langle L_{z} \right\rangle=∯ \boldsymbol{r}\times\mathbf{P}d\boldsymbol{r=}∯ \boldsymbol{r}\times\frac{\rho}{2\omega}\mathrm{Im}\left[ \mathbf{v}^{*}\cdot\left( \nabla\right)\mathbf{v} \right]d\boldsymbol{r}$, (S15)

$\left\langle S_{z} \right\rangle=∯ S_{z}d\boldsymbol{r=}∯ \frac{\rho}{2\omega}Im\left( \mathbf{v}^{*}\times\mathbf{v} \right)d\boldsymbol{r}$, (S16)

where $\mathbf{P}$ are the canonical momentum density. The normalized values of $\left\langle L_{z} \right\rangle$ (orange line) and $\left\langle S_{z} \right\rangle$ (blue dots) as functions of $\theta$ are shown in Figure S6a. $\left\langle L_{z} \right\rangle$ evolves in a manner consistent with $\left\langle S_{z} \right\rangle$, which indicates the synergy between the OAM and SAM. Additionally, acoustic pressure fields $\left| P \right|$, phase $arg\left( P \right)$, OAM and SAM density distributions for $\theta=\frac{\pi}{2}$(topological charge $l=1$) and $\frac{3\pi}{2}$ (topological charge $l=-1$) are respectively shown in Figure S6b and c, illustrating the synergy between OAM and SAM.

Furthermore, this synergy relationship between scalar acoustic pressure and vector acoustic velocity can even be extended to electromagnetic waves. For instance, in uniform media, transverse-magnetic waves also follow a gradient relationship between the vector magnetic field $\boldsymbol{H}$ and the scalar electric field$E_{z}$, which is expressed as $\boldsymbol{H}=-\frac{i}{\mu\omega}\nabla E_{z}$($\mu$ is the permeability).

**6.2 SAM** **and OAM density distributions in vortexes** **based on** $\boldsymbol{d}$-**orbitals.**


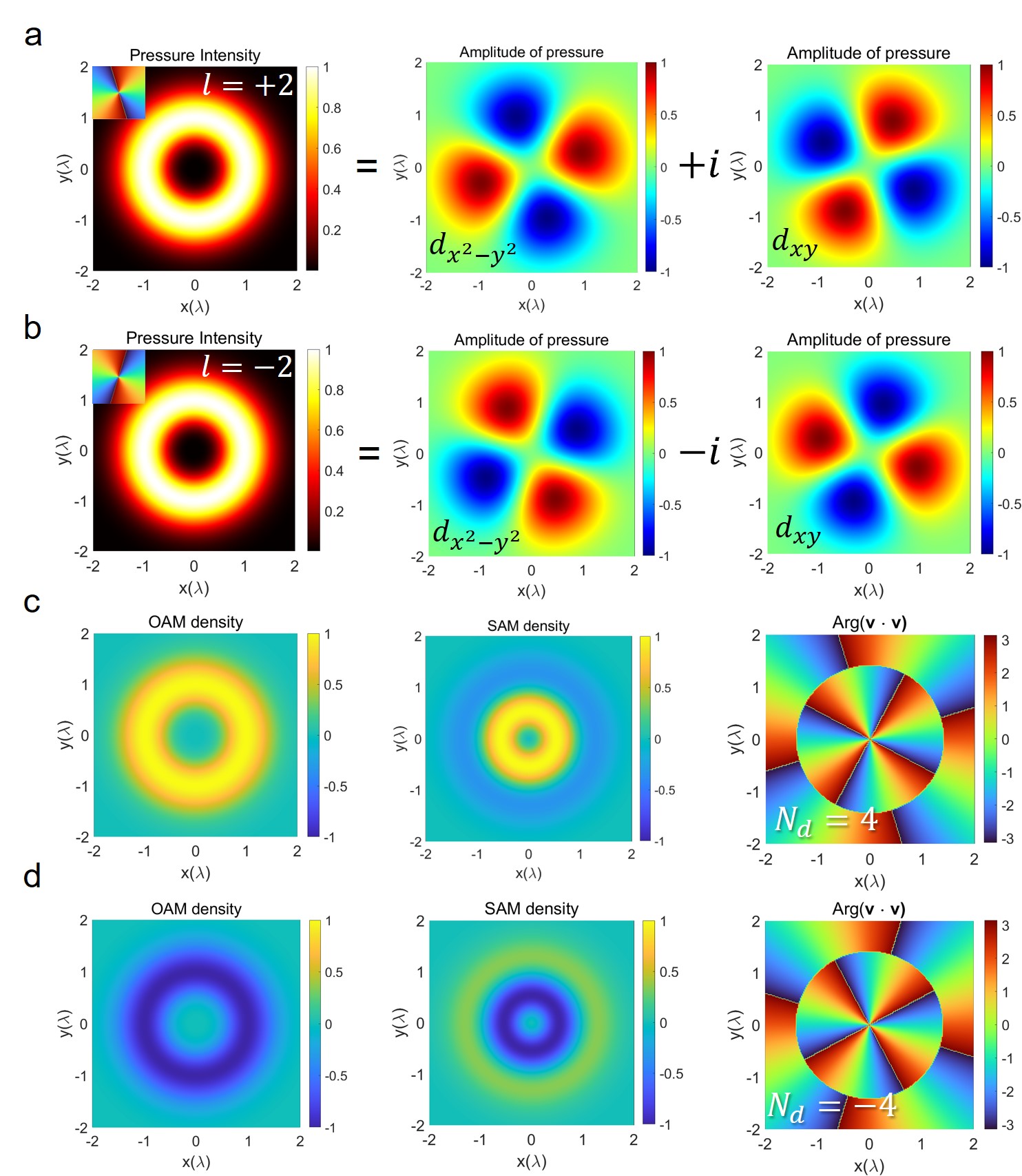


Figure S7. **a**, **b**, Left planes: acoustic pressure fields $\left| P \right|$, phase $arg\left( P \right)$ for vortexes with topological charges $l=\pm2$, respectively. Middle planes: Real parts of $P$, and they are $d_{x^{2}-y^{2}}$-orbitals-like states. Right planes: Image parts of $P$, and they are $d_{xy}$-orbitals-like states. **c**, OAM and SAM density, and the phase of complex scalar field$\arg\left( \Psi\right)$ distributions for vortex with topological charge $l=2$. **d**, The same as **c**, only for vortex with topological charge $l=-2$. ($z=5\lambda$)

Our model focuses on $p$-orbitals, leading to the evolution of orbital and polarization states within the vortexes possessing topological charges of$\pm1$. To demonstrate the applicability of our principle to a broader selection of orbitals, we analytically construct vortexes with topological charges of $\pm2$ based on $d$-orbitals. We start by considering vortexes with topological charges $l=\pm2$. According to Equation (S13), these are expressed as

$P\left( x,y \right)={P_{0}\left( x+i\cdot sign\left( l \right)\cdot y \right)}^{\left| l \right|}e^{-\frac{x^{2}+y^{2}}{\lambda^{2}}}e^{ikz}$. (S17)

The acoustic pressure fields $\left| P \right|$ and phase $arg\left( P \right)$ distributions for the topological charges $l=\pm2$ are shown in the left planes of Figure S7a and b, respectively. The fields can be decomposed into two parts: $real\left( P \right)$ and $imag\left( P \right)$, as shown in the right planes of Figure S7a and b. Obviously, the vortexes with topological charges $l=\pm2$ can be regarded as superpositions of $d$-orbitals, specifically $d_{x^{2}-y^{2}}\pm id_{xy}$. The phase difference between these two orthogonal $d$-orbitals is essential for generating OAM, which can be induced through periodic modulation in our principle, as discussed in the main text.

Furthermore, we examine the OAM and SAM density distributions following Supplementary Section 6.1. Figure S7c and d illustrate these distributions for vortexes with $l=\pm2$, respectively. The sign of SAM density aligns with that of the OAM, indicating the synergy between them. More intriguingly, we calculate the phases of the complex scalar fields of vector velocity $\Psi=$ $\mathbf{V}\boldsymbol{\cdot}\mathbf{V}$,^[7]^ as shown in the right planes of Figure S6c and d. These fields exhibit phase singularities with topological charges $N_{d}=\frac{\oint\nabla\arg\left( \Psi\right)d\boldsymbol{r}}{2\pi}=\pm4$, respectively, which are double the values of topological charges $l$ of pressure vortexes. This relationship also holds for vortexes with higher topological charges, as discussed in Supplementary Section 6.3, and is a manifestation of synergy between OAM and SAM in vortexes with various topological charges.

**6.3 SAM and OAM density distributions in vortexes with higher-order topological charges.**


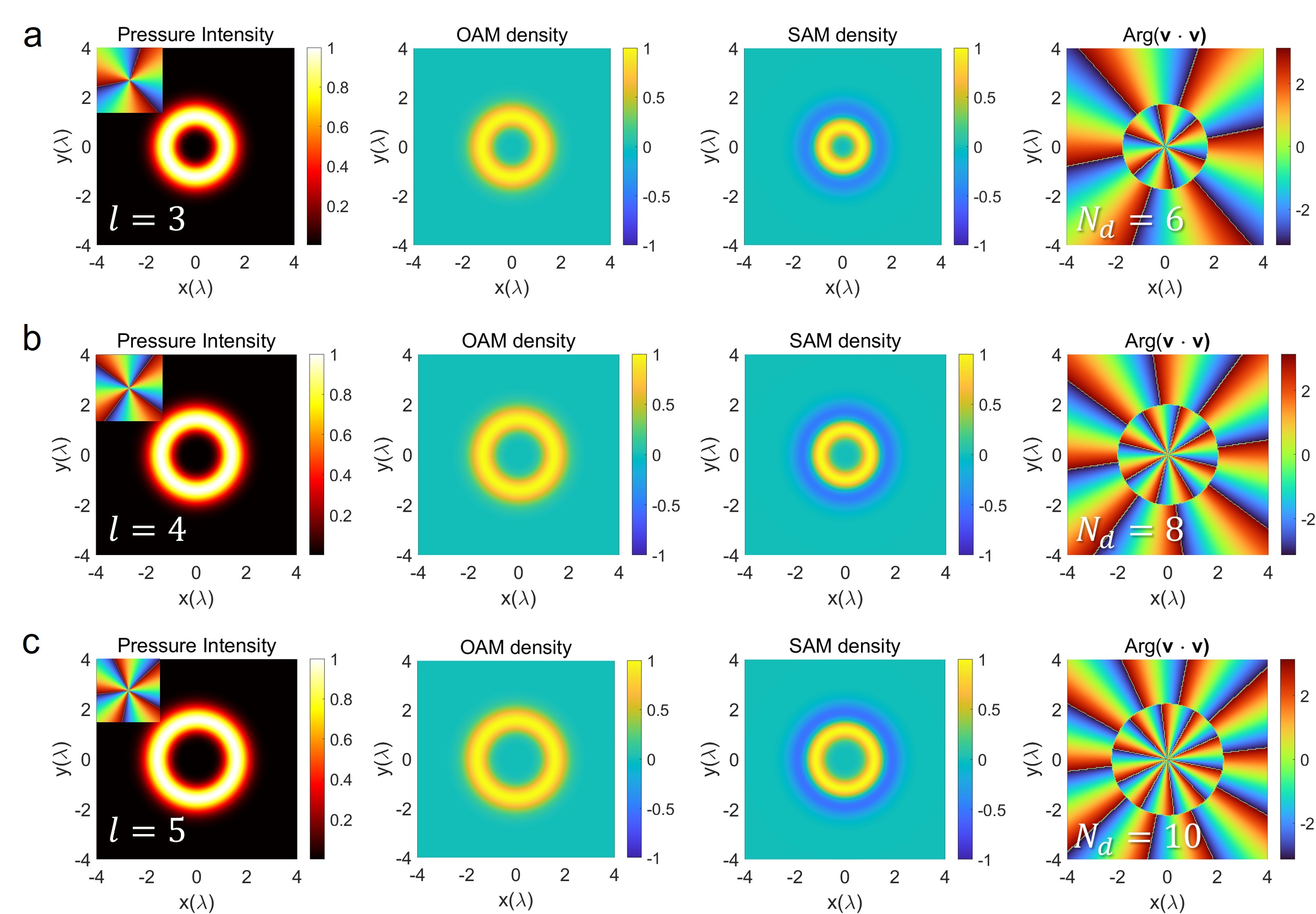


Figure S8. From left to right planes: acoustic pressure fields $\left| P \right|$, OAM density, SAM density and phase of complex scalar field $arg\left( \Psi\right)$ distributions for vortexes with topological charges $l=$3 (**a**), 4 (**b**) and 5 (**c**), respectively. The insets of left planes are $arg\left( P \right)$ distributions. ($z=5\lambda$)

Figure S8a, b, and c illustrate the phase distributions of the acoustic pressure fields $\left| P \right|$, along with the distributions of OAM and SAM density, as well as the phase distributions of the complex scalar field $\Psi$ for vortexes with topological charges $l=3$, $4$ and 5 respectively. Notably, the topological charge of the complex scalar field Ψ is twice that of the acoustic pressure fields $P$ in each of these cases. These observations underscore the synergy between OAM and SAM in vortexes with different topological charges.

**6.4 Evolution of synergized spin and orbital angular momenta based on** $\boldsymbol{d}$**-orbitals.**


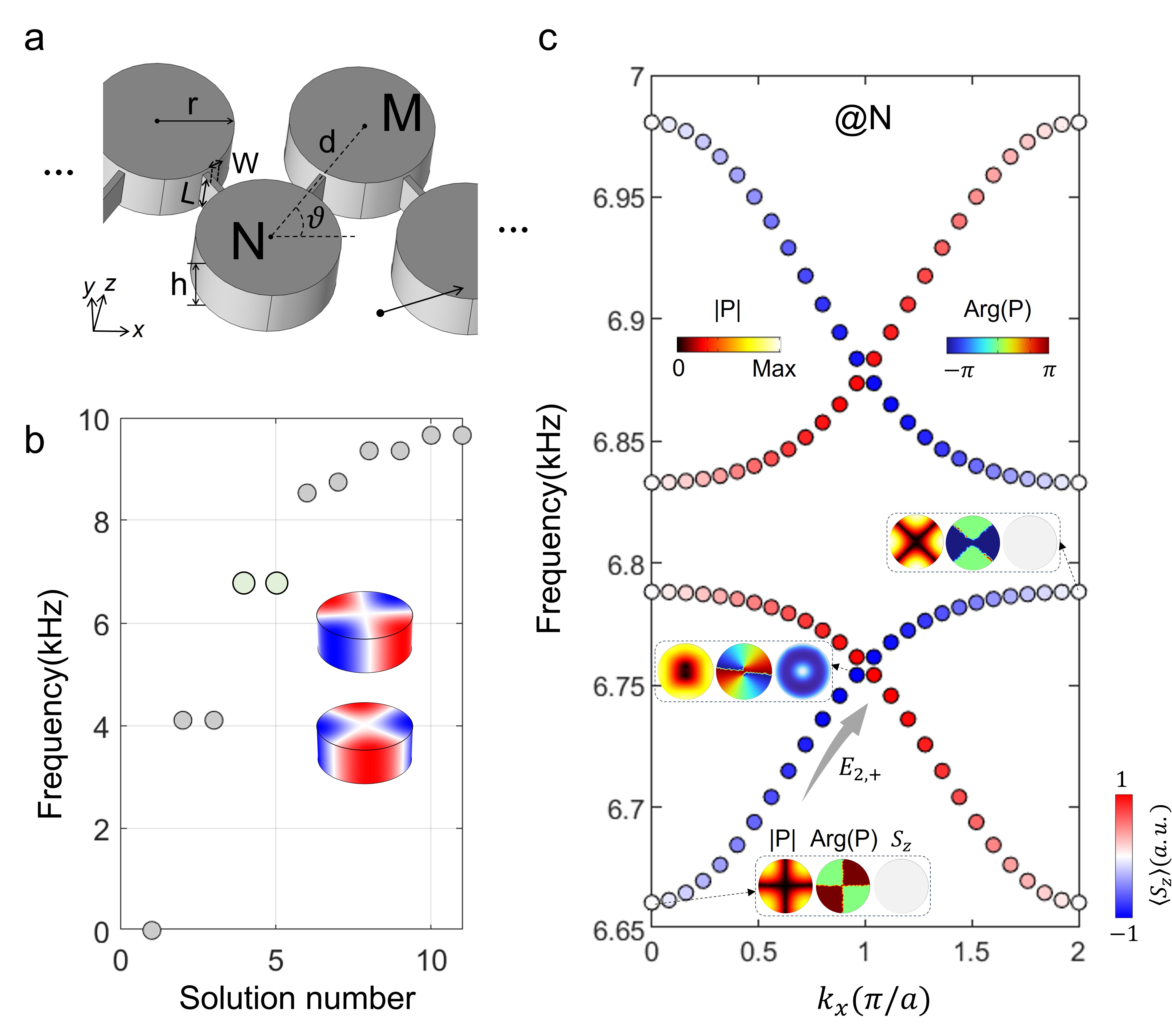


Figure S9 **a**, Schematics of the acoustic lattice. The geometric parameters are set as $d=5.88$ cm, $\vartheta=55^{\circ}$, $W=0.25 cm$, $L=1.57 cm$, $r=2.45$ cm and $h=1.96$ cm. **b**, Calculated eigenmodes and pressure field distributions for the acoustic cavity shown in (a). **c**, Band structures of the acoustic lattice in (a), with $\left\langle S_{z} \right\rangle$ color-coded for the cavity N. Here, $\left\langle S_{z} \right\rangle$ denotes the integration of the SAM density $S_{z}$ over the cavity N, marking its polarization evolution. The insets show the acoustic pressure fields $\left| P \right|$, phase distributions $\text{Arg}\left( P \right)$, and SAM density distributions $S_{z}$ for three evolution states along the $E_{2,+}$ band.

Our principle is universal and can be extended to a broad selection of orbitals, including $d$-orbitals and beyond. In Figure S9, we provide an example with $d$-orbitals. Figure S9a illustrates the acoustic lattice design, similar to the structure used in the main text, with optimized geometric parameters to isolate the $d$-orbital energy bands (by optimizing, it is also allowed for independent realization of $p$-, $d$- and even higher-orbital-based SAM-OAM synergy in a single lattice). Figure S9b presents the eigenmodes of each acoustic cavity, highlighting two degenerate quadrupole resonances at 6.8 kHz, mimicking the $d$-orbitals.

Following the analyses in the main text, we observe that the $d$-orbital energy bands also exhibit a synergized relationship between SAM and oam, as shown in Figures S9c. For clarity, only the evolution in cavity N is depicted. Along the $E_{2,+}$ band, a $d$-orbital first evolves into a perfect vortex, then transforms back to the $d$-orbital, but with a different orientation. Accompanying the orbital change, the SAM density transitions from zero to nonzero and then back to zero. This is consistent with the SAM-OAM evolution for $p$-orbitals as discussed in the main text, supporting the general applicability of our principle.

Interestingly, the $d$-orbital-based SAM-OAM evolution enables the manipulations of higher-order angular momenta, producing vortices with topological charges $l=\pm2$ and ripple-like distributions in the SAM density, demonstrating rich SAM-OAM interactions.

**6.5 Evolution of synergized spin and orbital angular momenta in photonic system.**


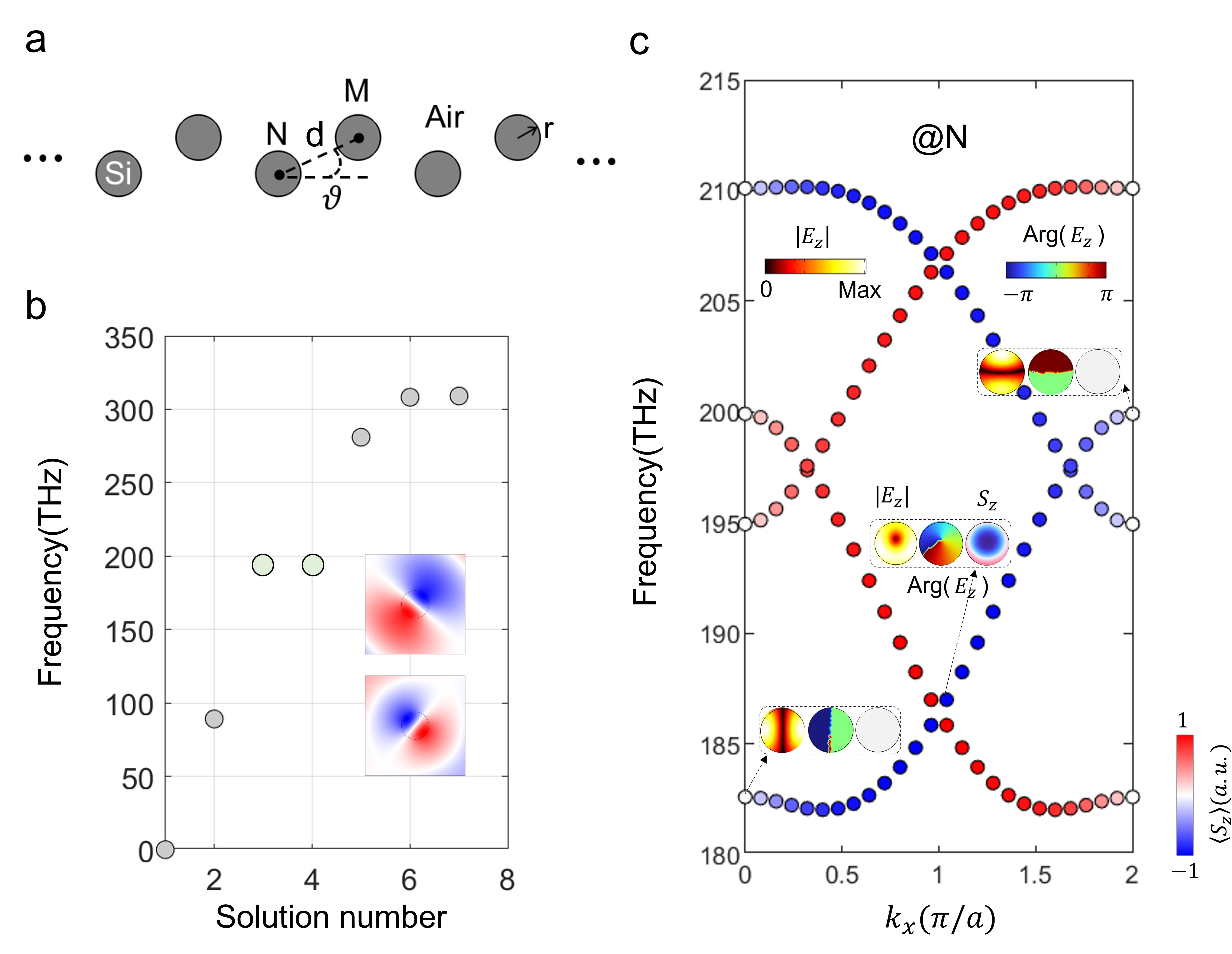


Figure S10 **a**, Schematics of a quasi-1D photonic lattice. **b**, Eigenspectrum of a single silicon rod in air, with two dipole resonances highlighted. **c**, Band structures with $\left\langle S_{z} \right\rangle$ color-coded for rod N. The insets show the electric fields $\left| E_{Z} \right|$, phase $\text{Arg}\left( E_{Z} \right)$, and SAM density distributions $S_{z}$.

Our principle is also applicable to photonic systems. As an example, we design a quasi-1D photonic crystal consisting of silicon rods ($\epsilon_{\mathrm{silicon}}$ = 11.8) in air, as shown in Figure S10a. The geometric parameters are set as $d=642$ nm, $\vartheta=40^{\circ}$, and $r=165$ nm. The eigenspectrum of each silicon rods is presented in Figure S10b, where two degenerate dipole resonances emerge at 193 THz, mimicking the $p$-orbitals. The energy bands are shown in Figure S10c, which again exhibit SAM-OAM synergized evolution, only now the scalar filed is represented by the z-component of the electric field for a TM-wave while the vector field corresponds to the magnetic field. When considering 3D cases where both the electric and magnetic fields are fully vectorial, more complex and intriguing SAM-OAM interactions are expected (which we aim to explore in future work).

1. **Measured SAM states on polarization Poincaré sphere and spin density at different positions.**


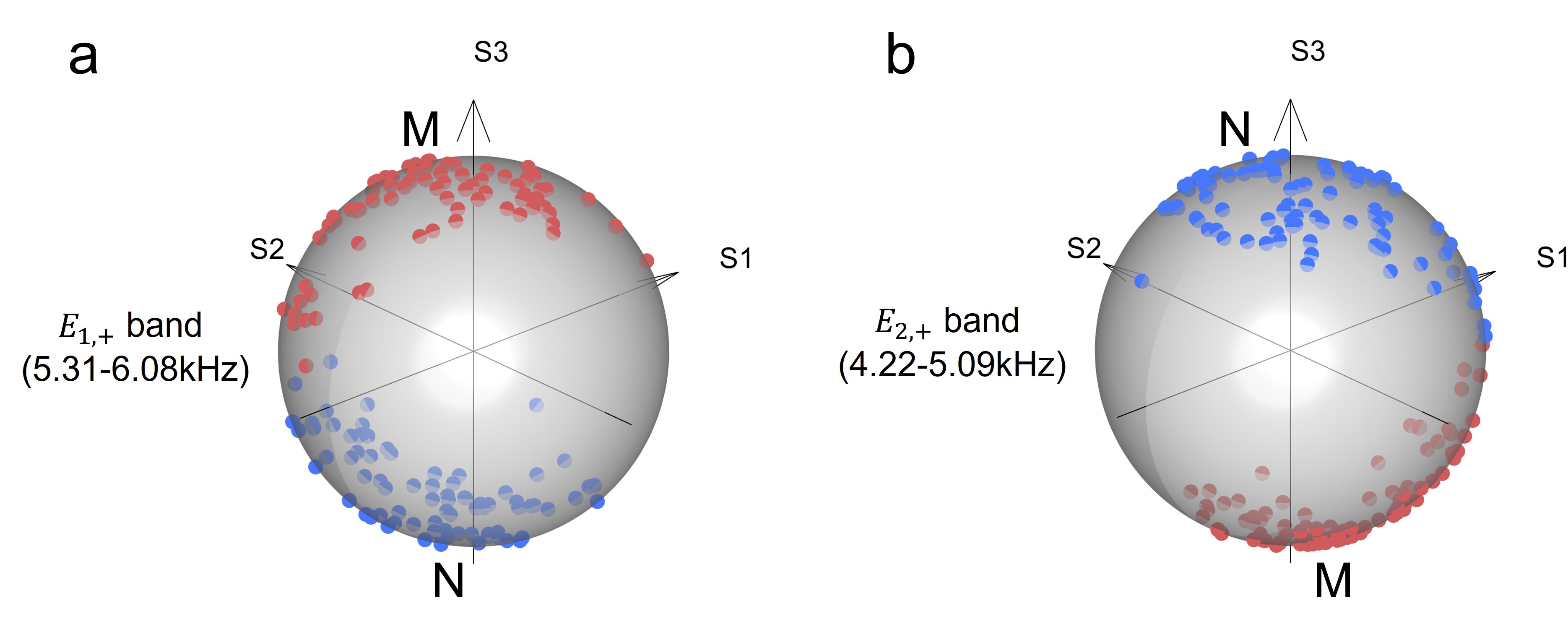


Figure S11 **a**, SAM states on Polarization Poincaré sphere for the $E_{1,+}$ band. **b**, The same as (a), only for $E_{2,+}$ band.

Here, we present the measured SAM states on the Poincaré sphere, parameterized by the Stokes parameters $\{S_{1}/S_{0},S_{2}/S_{0},S_{3}/S_{0}\}$, where $\{S_{0},S_{1},S_{2},S_{3}\}=\{\left| V_{x} \right|^{2}+\left| V_{y} \right|^{2},\left| V_{x} \right|^{2}-\left| V_{y} \right|^{2},2\mathrm{Re}(V_{x}\cdot{V_{y}}^{*}), -2\mathrm{Im}(V_{x}\cdot{V_{y}}^{*})\}$. It is observed that for the $E_{1,+}$ band, the SAM states at Site M are located in the northern hemisphere, while those at Site N are in the southern hemisphere. For the $E_{2,+}$ band, the locations of the SAM states are reversed. These observations are consistent with both the theoretical predictions and experimental measurements shown in Figure 3 of the main text.

To further verify the synergy between SAM and OAM, we experimentally measure the SAM at specific points within cavities M and N. These points, labeled P1, P2, P3, P4 and center, are illustrated in the inset of Figure S12a. The experimental setup for these measurements is identical to that used in the main text. Following the data collection, we calculate the normalized spin density following $\mathbf{S}=\frac{\rho}{2\omega}\mathrm{Im}\left( \mathbf{V}^{*}\times\mathbf{V} \right)$. The results for points P1, P2, P3, P4 and the center of cavity M, when $k>0$ and $k<0$, are shown in Figure S12a and b, respectively. The spin densities at P1, P2, P3 and P4 points are consistent with that at the center. Additionally, the spin density for $k>0$ is reversed compared to that for $k<0$. In cavity N, as shown in Figure S12c and d, the spin densities at P1, P2, P3 and P4 points are also in agreement with that at the center. Moreover, the spin densities under excitation with $k>0$ and $k<0$ exhibit a reversal sign when compared to those in cavity M. These alignments corroborate the synergy between SAM and OAM.


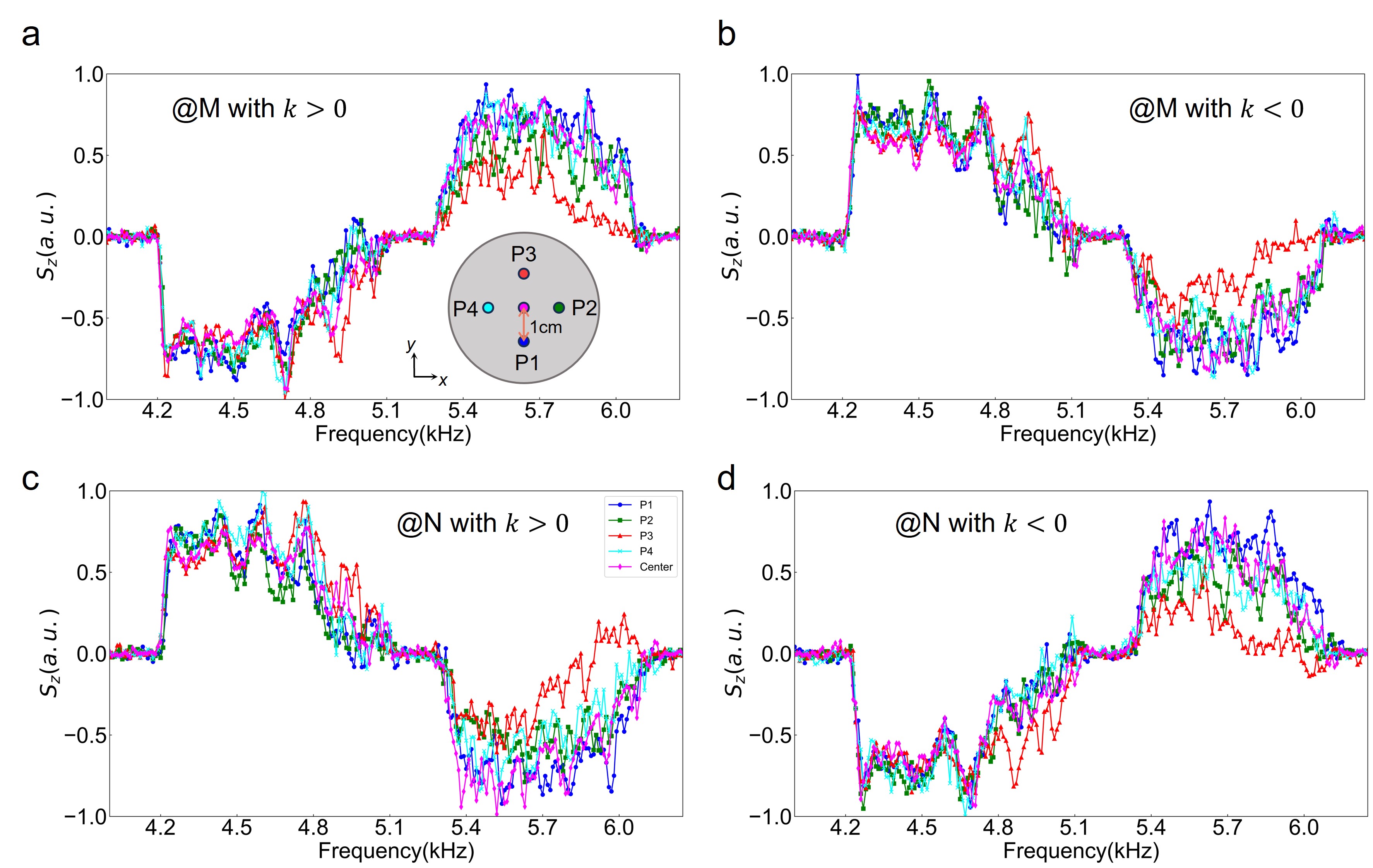


Figure S12. Spin densities for different points in cavities M and N. **a**, **b,** Spin densities for cavity M with $k>0$ and $k<0$, respectively. **c**, **d**, The same as **a** and **b**, only for Site N. The inset in **a** shows the positions of points P1, P2, P3 and P4 in the *x-y* plane, located 1 cm from the center of the cavity.

1. **Influences of the wide coupling tube.**


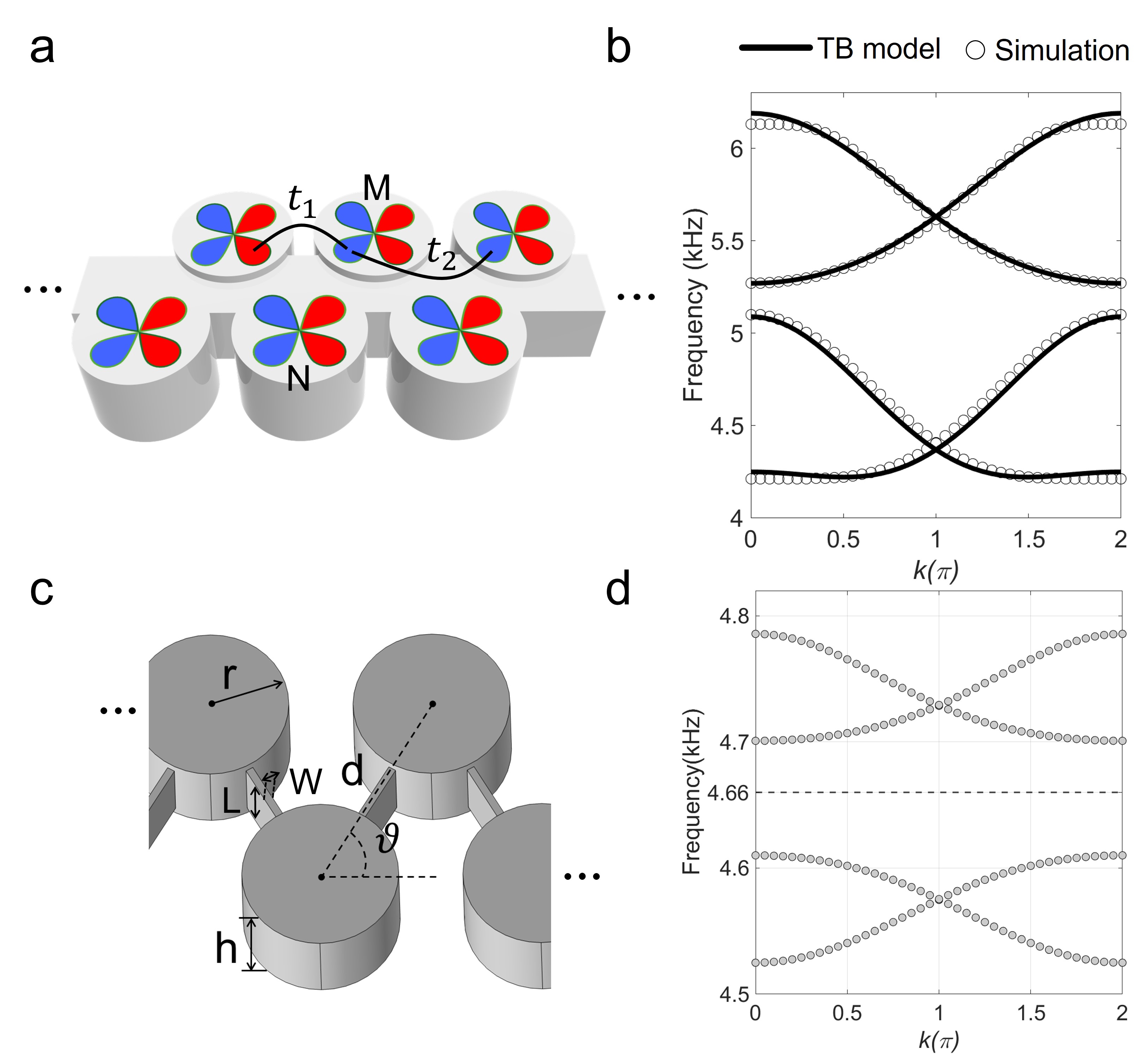


Figure S13. **a**, Schematics of the long-range couplings. **b**, Comparison of energy bands between the fitted TB model (black lines) and the acoustic lattice (dots). **c**, Schematics of a quasi-1D acoustic lattice with smaller coupling tubes. The geometric parameters are taken as $d=6.08$, $\vartheta=60^{\circ}$, $W=0.25 cm$, $L=1.57 cm$, $r=2.16$ cm and $h=2.75$ cm. **d**, Band structures of the acoustic lattice shown in (c).

In an ideal scenario with chiral symmetry, the four bands would be symmetric around the resonant frequency. However, in our designed acoustic lattice, the resonant frequency deviates from the band center due to two primary reasons: 1) The size of the coupling tube is comparable to that of the cylindrical cavity, leading to a shift in the resonant frequency; 2) The relatively wide coupling tube introduces long-range couplings, which slightly break the chiral symmetry and further shift the center frequency. To provide concrete evidence, we refine the tight-binding (TB) model to account for both the resonant frequency shift and long-range couplings, as illustrated in Figure S13a. The Hamiltonian, corresponding to the eigenfunction in the main text, is given by

$H(k)=\left[ \begin{matrix} \Omega+t_{2}e^{-ik}+t_{2}e^{ik} & t_{1}e^{-ik} & t_{t}+t_{l}e^{ik} & t_{m}+t_{m}e^{ik} \\ t_{1}e^{ik} & \Omega+t_{2}e^{-ik}+t_{2}e^{ik} & t_{m}+t_{m}e^{ik} & t_{l}+t_{t}e^{ik} \\ t_{t}+t_{l}e^{-ik} & t_{m}+t_{m}e^{-ik} & \Omega+t_{2}e^{-ik}+t_{2}e^{ik} & t_{1}e^{ik} \\ t_{m}+t_{m}e^{-ik} & t_{l}+t_{t}e^{-ik} & t_{1}e^{-ik} & \Omega+t_{2}e^{-ik}+t_{2}e^{ik} \end{matrix} \right]$, (S18)

where $\Omega$ is the shifted resonant frequency, and $t_{1}, t_{2}$ represent the long-range couplings. Based on the simulated band structures, the parameters $\Omega,t_{1}, t_{2},$ together with the original nearest-neighbor couplings $t_{t}, t_{m}, t_{l}$, are fitted as $5.1$ kHz, $-0.02$ kHz, $0.05$ kHz, $-0.05$ kHz, $0.22$ kHz, $-0.58$ kHz, respectively. As shown in Figure S13b, the energy bands calculated from the fitted TB model agree well with the simulation results. Notably, the fitted long-range couplings are much smaller than the nearest-neighbor couplings, indicating that the coupling tube primarily shifts the resonant frequency. This shift only affects the frequencies of the SAM and OAM states, but has minimal impact on the states themselves, which are predominantly governed by the couplings between the$p$-orbitals.

As complementary evidence, we further design an acoustic lattice with smaller coupling tubes, as depicted in Figure S13c. The acoustic cavities remain unchanged, with the two degenerate dipole resonances at 4.66 kHz. As the influences induced by the coupling tubes are diminished, the resonant frequency lies at the band center, restoring chiral symmetry, as illustrated in Figure S13d. However, the narrower coupling tubes reduce the frequency window, posing challenges for experimental implementation. Therefore, in the main text, we opted for a wider coupling tube to achieve a broader bandwidth and to minimize scattering during sound wave propagation. Importantly, despite the breaking of chiral symmetry, the SAM-OAM evolution and their synergized relationship remain unaffected, as explained above.

1. **Full cycles of M- and N-evolutions**


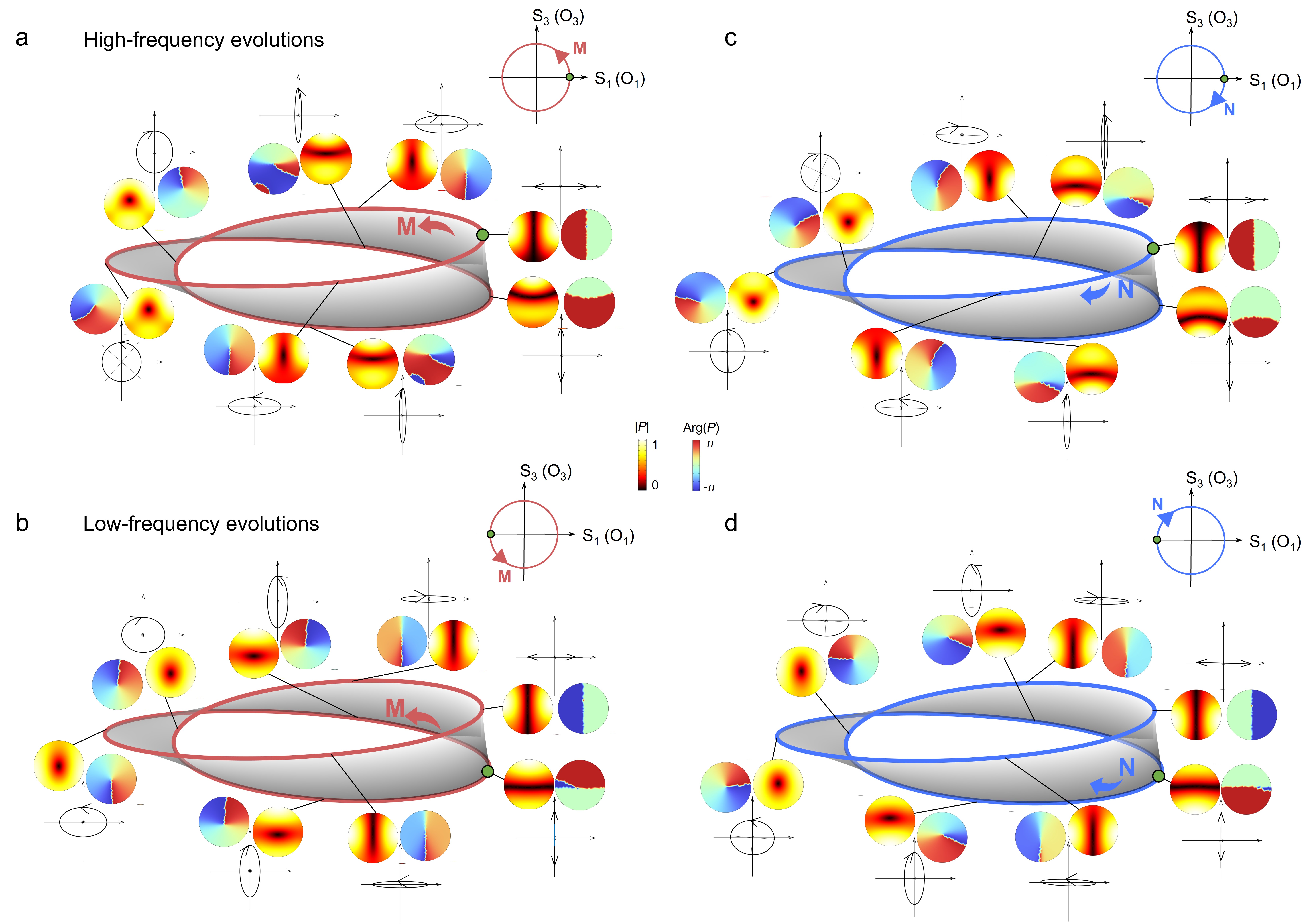


Figure S14. Full cycles of orbital and polarization states on cavities M and N. The pressure fields and their phase distributions are as spatial OAM features and the polarization ellipses based on the velocity fields at the center of cavity are as vector polarization features. The evolutions of orbital and polarization states at both high- and low-frequency bands are presented. Eigenstates in each band correspond to $k$ values being $0\left( \pi/a \right)$, 0.4$\left( \pi/a \right)$, 0.95$\left( \pi/a \right)$, 1.6$\left( \pi/a \right)$ and 2$\left( \pi/a \right)$. The starting point of each evolution is marked by a green dot, and the directions of evolutions are indicated by arrows.

As discussed in Supplementary Section 3, the evolutions of states on Sites M and N occur in opposite directions. Therefore, a combined half-cycle from both M- and N- evolutions constitutes a full cycle, as elaborated in the main text. Here, comprehensive demonstrations of the full cycles of orbital and polarization states for both cavities are shown in Figure S14. Figure S14a illustrates the evolution for states along the $E_{1,+}$ and $E_{1,-}$ bands on cavity M. Specifically, for $E_{1,+}$ band, an *x*-oriented $p$-orbital with horizontal linear polarization (green dot) transitions into a vortex with $l=+1$ and right-circular polarization, then transforms into a *y*-oriented $p$-orbital with vertical linear polarization. Continually, in the $E_{1,-}$ band, the *y*-oriented $p$-orbital with vertical linear polarization evolves into a vortex with $l=-1$ and left-circular polarization, eventually returns to an *x*-oriented $p$-orbital with horizontal linear polarization. This sequence of changes constitutes a full cycle for the M- evolution. Similarly, the N-evolution for $E_{1,+}$ and $E_{1,-}$ bands, as shown in Figure S14c, mirrors the M-evolution but in the reversed direction. Thus, the amalgamation of M- and N- evolutions for the same band completes a full cycle, equivalent to either an entire M- or N- evolution. The same principle extends to the low-frequency $E_{2,+}$ and $E_{2,-}$ bands for cavities M and N, as shown in Figure S14b and d, respectively.

1. **Relation between** **spin density and directional contrast**

The directional emission of the chiral source can be described by summing two coupling efficiencies for opposite handedness of the local chirality of velocity fields. Consider right- and left-handed circular sources on the *x-y* plane, defined as $\mathbf{s=}\frac{1}{\sqrt{2}}\left( \hat{\boldsymbol{x}}\pm i\hat{\boldsymbol{y}} \right)$, or equivalently, $\frac{1}{\sqrt{2}}\left[ \begin{matrix} 1 & \pm i \end{matrix} \right]^{T}$. For each position in the *x-y* plane of the fields, the guided eigenmodes can be decomposed into two elliptical polarizations associated velocity modes propagating in opposite directions as

$\mathbf{V}_{f\left( b \right)}\boldsymbol{=}\frac{1}{\sqrt{v_{x}^{2}+v_{y}^{2}}}\cdot\left( V_{x}\hat{\boldsymbol{x}}+V_{y}e^{+(-)i\Delta\zeta}\hat{\boldsymbol{y}} \right)$, (S19)

where $\Delta\zeta$ is the phase difference between $V_{x}$ and $V_{y}$ components. The emission rates of the circular chiral sources into the two opposite-handedness of elliptical modes are given by^[8]^

$\Gamma_{f\left( b \right)}\propto\left| \mathbf{s}^{\boldsymbol{*}}\boldsymbol{\cdot}\mathbf{V}_{f\left( b \right)} \right|^{2}=\left| \frac{1}{\sqrt{2}}\left( \hat{\boldsymbol{x}}\pm i\hat{\boldsymbol{y}} \right)^{\boldsymbol{*}}\boldsymbol{\cdot}\frac{1}{\sqrt{V_{x}^{2}+V_{y}^{2}}}\cdot\left( V_{x}\hat{\boldsymbol{x}}+V_{y}e^{+(-)i\Delta\zeta}\hat{\boldsymbol{y}} \right) \right|^{2}$. (S20)

The directional contrast is then defined as

$\Gamma=\frac{\Gamma_{f}-\Gamma_{b}}{\Gamma_{f}+\Gamma_{b}}=\frac{2V_{x}V_{y}\sin\mp\Delta\zeta}{V_{x}^{2}+V_{y}^{2}}$. (S21)

Furthermore, based on the definition of spin density, we have

$S_{z}\propto\mathrm{Im}\left( {\mathbf{V}_{f\left( b \right)}}^{*}\times\mathbf{V}_{f\left( b \right)} \right)=\frac{2V_{x}V_{y}\sin\mp\Delta\zeta}{V_{x}^{2}+V_{y}^{2}}$. (S22)

Therefore, the directional contrast of circular chiral source emission is directly proportional to the local spin density of velocity fields.

1. **Field distributions under excitations by different chiral sources**


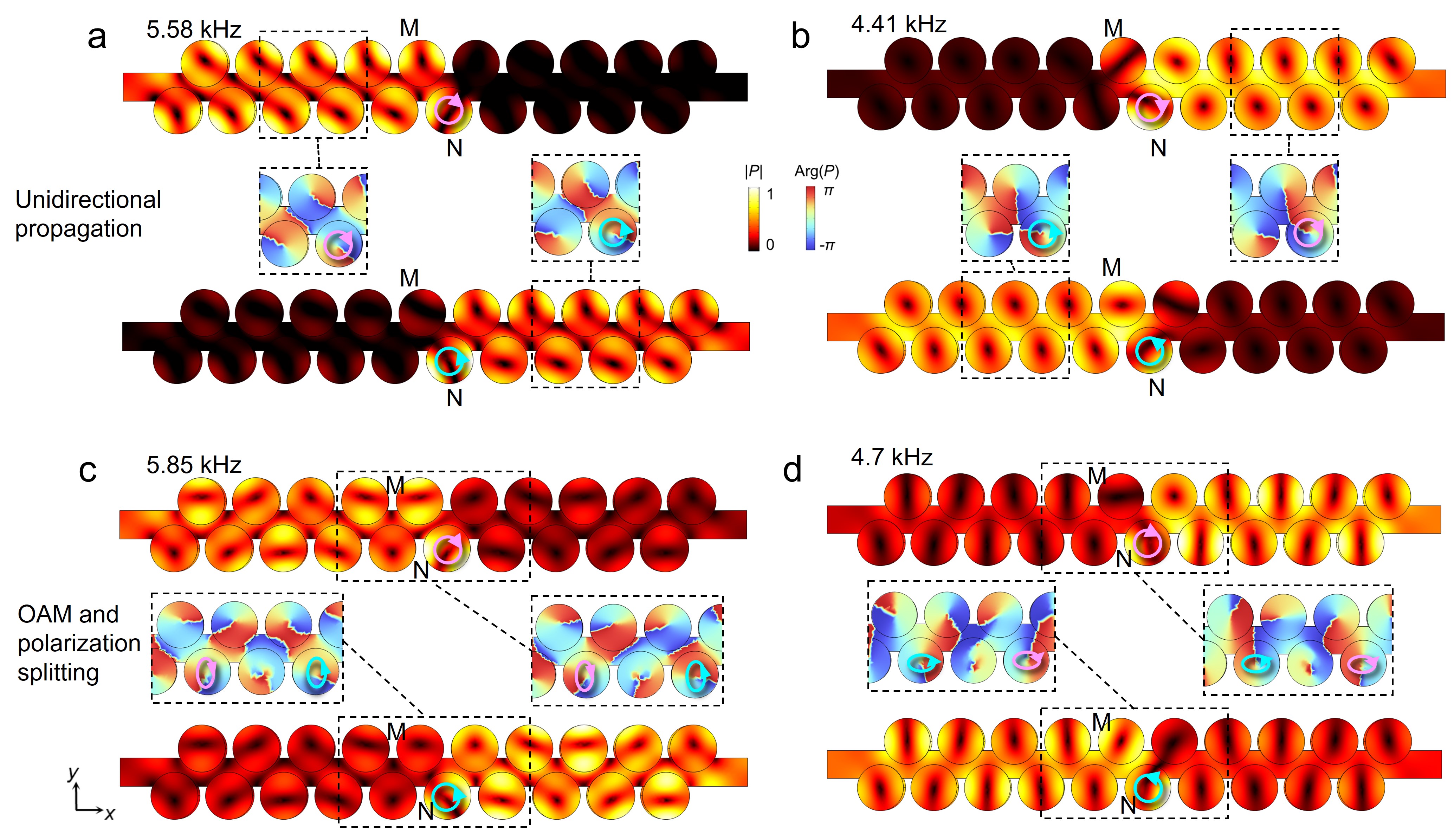


Figure S15. **a**, **b,** Pressure fields and their phase distributions at excitation frequencies of 5.58 and 4.41kHz (precisely corresponding to the poles accommodating circular polarizations), respectively. In each panel, the top corresponds to the case with excitation by a right-handed chiral source (pink arrow), while the bottom represents excitation by a left-handed chiral source (light blue arrow). **c**, **d**, The same as **a**, **b**, only for excitation frequencies of 5.85 kHz and 4.7 kHz.

In our system, the SAM and OMA are synergized, suggesting a tunable polarization- and OAM-splitting. Employing the same excitation conditions as in Figure 4, we placed a circular chiral source in cavity N to excite propagating modes. The simulated pressure fields and their phase distributions are shown in Figure S15. In Figure S15a and b, the distributions are presented at excitation frequencies of 5.58 kHz and 4.41 kHz, respectively. The top and bottom planes are the cases excited by right- and left-handed chiral sources, respectively. Since the eigenstates are perfect OAM states with topological charges of $\pm1$, the energies from the chiral sources are almost entirely coupled into the corresponding chiral eigenstates, resulting in unidirectional propagations. However, in the case of imperfect OAM states, the sources decompose into two eigenmodes propagating in opposite directions. The efficiencies of energy coupling are determined by the compatibility between the source and the propagating modes. At excitation frequencies of 5.85 kHz and 4.7 kHz, as shown in Figure S15c and d, the energies of sources are coupled into two imperfect OAM states, with a large portion transferred into the preferred-handedness direction and a small portion into the opposite direction with reversed-handedness. As the guided eigenmodes evolve, the tunable polarization- and OAM-splitting is achieved.

**Supplementary movies**

**Movie S1.**

The vector velocity fields (at the middle $x$-$y$ plane of the cavities) are time-varying at a frequency of 4.41 kHz (with $k$ close to $\pi/a$). The velocity fields exhibit left-handed circular polarization for cavity M (left) and right-handed circular polarization for cavity N (right).

**Movie S2.**

The vector velocity fields (at the middle $x$-$y$ plane of the cavities) are time-varying at a frequency of 4.41 kHz (with $k$ close to $-\pi/a$). The velocity fields exhibit right-handed circular polarization for cavity M (left) and left-handed circular polarization for cavity N (right).

**References**

1. G. Cáceres-Aravena, L. F. Torres, and  R. A. Vicencio, Topological and flat-band states induced by hybridized linear interactions in one-dimensional photonic lattices, *Phys. Rev. A* **102**, 023505 (2020).
2. J. Schulz, J. Noh, W. A. Benalcazar, G. Bahl, G. von Freymann, Photonic quadrupole topological insulator using orbital-induced synthetic flux. *Nat. Commun.* **13**, 6597 (2022).
3. S. M. Barnett, Optical angular-momentum flux. *J. Opt. B: Quantum Semiclassical Opt.* **4**, S7 (2001).
4. D. Vanderbilt, *Berry phases in electronic structure theory: electric polarization, orbital magnetization and topological insulators* (Cambridge University Press, Cambridge, England, 2018).
5. A. P. Slobozhanyuk, A. N. Poddubny, A. E. Miroshnichenko, P. A. Belov, Y. S. Kivshar, Subwavelength topological edge states in optically resonant dielectric structures. *Phys. Rev. Lett.* **114**, 123901 (2015).
6. F. Gao, X. Xiang, Y.-G. Peng, X. Ni, Q.-L. Sun, S. Yves, X.-F. Zhu, A. Alù, Orbital topological edge states and phase transitions in one-dimensional acoustic resonator chains. *Nat. Commun.* **14**, 8162 (2023).
7. M. R. Dennis, K. O’holleran, M. J. Padgett, Singular optics: optical vortices and polarization singularities. *Prog. Opt.* **53**, 293 (2009).
8. S.-H. Gong, F. Alpeggiani, B. Sciacca, E. C. Garnett, L. Kuipers, Nanoscale chiral valley-photon interface through optical spin-orbit coupling. *Science* **359**, 443 (2018).
